# Microbiota composition of the female reproductive tract and miscarriage: a systematic review and meta-analysis

**DOI:** 10.1038/s41522-025-00901-9

**Published:** 2026-01-08

**Authors:** Naomi Black, Ian Henderson, Siobhan Quenby, Joshua Odendaal, David A. MacIntyre

**Affiliations:** 1 Division of Biomedical Sciences, Clinical Sciences Research Laboratories, Warwick Medical School, Tommy’s National Centre for Miscarriage Research, University of Warwick, Coventry, UK; 2 University Hospitals Coventry & Warwickshire, Coventry, UK; 3 National Perinatal Epidemiology Unit, University of Oxford, Oxford, UK; 4 Tommy’s National Centre for Miscarriage Research, Imperial College London, London, UK; 5 March of Dimes Prematurity Research Centre at Imperial College London, London, UK; 6 Imperial College Parturition Research Group, Institute of Reproductive and Developmental Biology, Department of Metabolism, Digestion and Reproduction, Imperial College London, London, UK; 7 Robinson Research Institute, University of Adelaide, Adelaide, Australia

**Keywords:** Next-generation sequencing, Health care

## Abstract

Miscarriage, the loss of a pregnancy before viability, can be sporadic or recurrent. Emerging evidence links miscarriage to specific microbiota compositions within the female reproductive tract (FRT). This systematic review aims to synthesise evidence on the association between sporadic and recurrent miscarriage and FRT microbiota composition, as assessed using metataxonomic profiling approaches. A systematic analysis of the 43 included studies, sampling the vaginal, cervical and endometrial microbiota supported an association between reduced *Lactobacillus* abundance and miscarriage, making it a potential target for therapeutic intervention. However, consistent changes in alpha and beta diversity were not observed and there was a lack of reproducibility for other compositional changes. This review also highlighted concerns about the significant bias introduced due to methodological variations and emphasises the need for future standardisation of microbial sampling, sequencing, and reporting to allow accurate comparison of results and to reduce research waste.

## Introduction

Miscarriage, defined as the spontaneous loss of pregnancy prior to viability, remains a distressing and common reproductive event, affecting 15% of clinically recognised pregnancies^1^. It can be subclassified as sporadic or recurrent. Sporadic miscarriage refers to pregnancy losses that occur unpredictably. While 50% of sporadic miscarriages are attributed to underlying fetal chromosomal abnormalities, the cause of the rest remains unexplained^2^. Recurrent miscarriage (RM) or Recurrent Pregnancy Loss (RPL) refers to the loss of two or more pregnancies and is considered a distinct condition^3^, with pregnancy loss occurring more frequently than by chance. Although the rate of chromosomal abnormalities remains constant in relation to the number of previous miscarriages, a higher order of losses is predictive of subsequent miscarriage^4^. Despite advances in this field, many cases of pregnancy loss remain unexplained and the search for underlying mechanisms continues.

The female reproductive tract (FRT) contains a dynamic community of microorganisms collectively termed the microbiota, including but not limited to bacteria, viruses and fungi^5^. More broadly, the microbiota, their metabolites, genetic material and host-microbiota interactions are known as the microbiome^5^. The FRT microbiome regulates key biological processes and disruptions in microbiota-host interactions have been associated with conditions including gynaecological malignancies^6–8^, infertility^9^, preterm birth^10–12^ and polycystic ovarian syndrome^13^. Colonisation of the vaginal microbiota begins at birth and is influenced by factors such as age^14,15^, ethnicity^16,17^, diet^18^, hygiene practices^19^ and the menstrual cycle^20,21^. At reproductive age, dominance of the vaginal microbiota by *Lactobacillus*, a gram-positive facultative anaerobe that produces lactic acid to lower pH, is a hallmark of vaginal health^17^. *Lactobacillus* is also a member of the endometrial microbiota, which is characterised by a significantly lower bacterial load and overall higher diversity than the vagina^22^. Displacement of *Lactobacillus* from the FRT by pathogenic bacteria is often considered a state of dysbiosis^23^ and can impact embryo implantation and pregnancy development. For example, pathogenic bacteria can drive aberrant local immune responses that negatively affect endometrial receptivity and fetal-maternal immune tolerance^24–26^.

The advent of next generation sequencing approaches has facilitated comprehensive detection and characterisation of the FRT microbiota (FRTM). In this systematic review, we aimed to synthesise existing evidence on the association between the FRTM and miscarriage, and examine methodological approaches and reporting biases across studies.

## Results

**Fig. 1:**
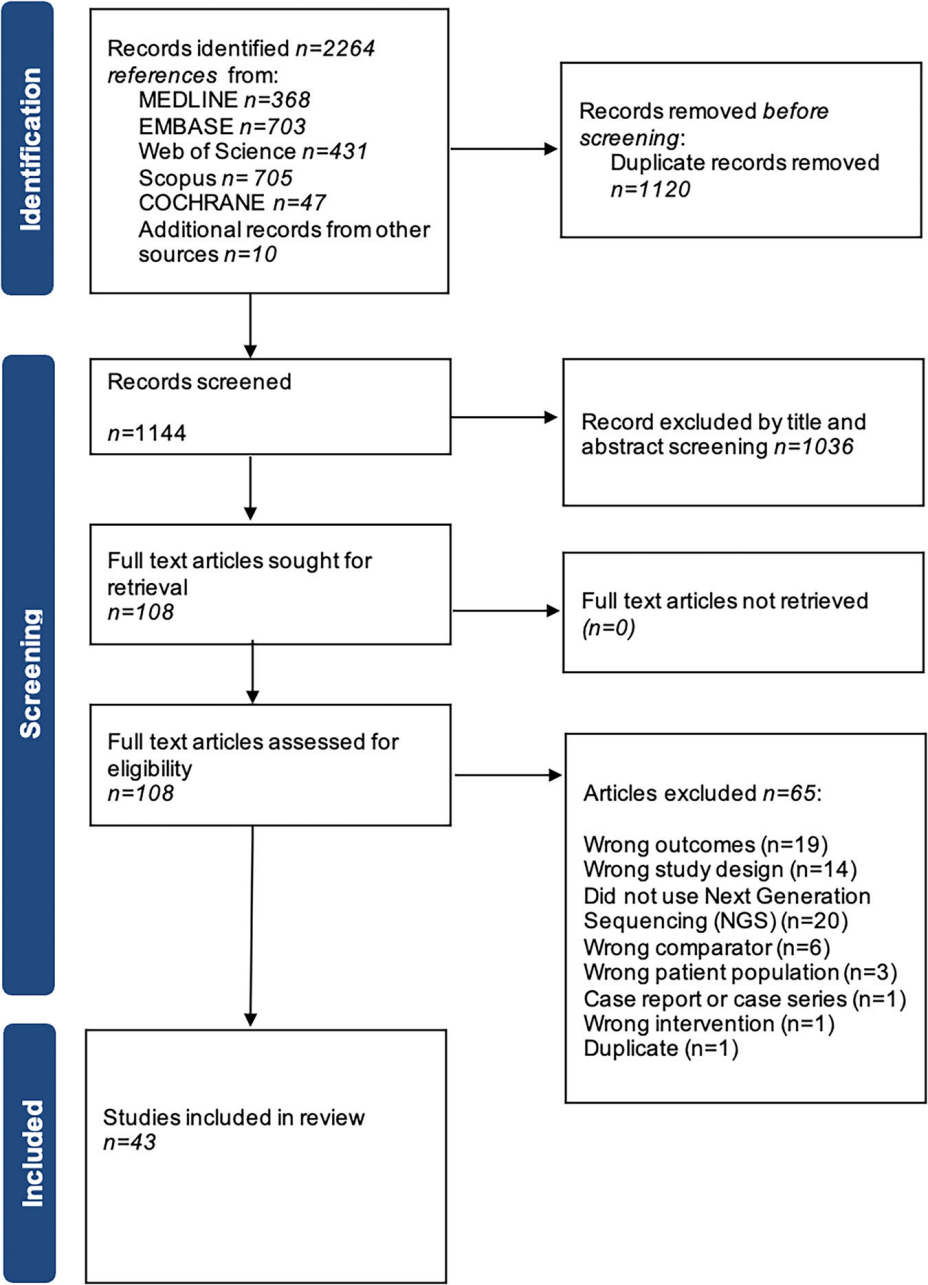
Preferred Reporting Items for Systematic Reviews and Meta-analysis (PRISMA) flow diagram summarising flow of studies in systematic review.

The search identified 2264 citations, with 1144 remaining after duplicate exclusion. Of these, 108 were assessed by full-text review and 43 were included as summarised in a PRISMA flow chart (Fig. 1).

### Characteristics of included studies

The 43 studies included 5064 participants: 1543 cases and 3521 controls. Total sample sizes ranged from 10 to 1984, with a median of 50 combined cases and controls. Study characteristics are presented in Table 1 and Supplementary Table 4. The studies were published between 2015 and 2025 and were from 13 countries, with most studies (*n* = 18) conducted in China. Several studies sampled more than one FRT site, specifically the vagina (*n* = 27)^27–54^, cervix (*n* = 6)^34,35,38,42,55,56^ and endometrium (*n* = 18)^38,39,41,43,50,57–69^. No other FRT site was investigated. Sporadic miscarriage was investigated by 26 studies and recurrent miscarriage by 17, with two studies investigating both. Recurrent miscarriage was defined as two or more consecutive miscarriages (*n* = 4 studies)^36,38,39,48^ two or more non-consecutive miscarriages (*n* = 6)^30,42,54,58,64,66^, three or more consecutive miscarriages (*n* = 2)^53,68^ three or more non-consecutive miscarriages (*n* = 3)^31,33,57^, three or more consecutive clinical first trimester losses or two if one occurred in the second trimester (*n* = 1)^43^ and finally three or more miscarriages or at least two if one was a euploid loss (*n* = 1)^67^.

**Table 1:** Overview of included studies

Author (Year)	Country	Sporadic (S) or recurrent miscarriage (R) population	Body site sampled	Case definition	Control definition	*N* (Cases) No. recruited (No. analysed)	*N* (Controls) No. recruited (No. analysed)	Matched variables	Alpha diversity metrics	Beta diversity metrics	Newcastle Ottawa score
Al-Memar^27^	UK	S	Vagina	First (<14 + 0/40) or second (14 + 1–24 + 0/40) miscarriage	Term pregnancy	64 (1st Trimester) 14 (13) (2nd Trimester)	83 (81)	Age, ethnic group, BMI	Inverse Simpson, Observed species	NR	8
Bai^57^	China	R	Endometrium	History of RPL (3+ consecutive), current miscarriage of previous viable pregnancy	Healthy controls, no history of miscarriage, undergoing elective TOP	10	28	–	Chao1, Shannon, Simpson	PCA: Bray–Curtis	4
Barinova^58^	Russia	R	Endometrium	History of RPL (2+)	Healthy fertile controls	14	15	–	NR	NR	4
Bui^59^	Netherlands	S	Endometrium	Miscarriage at pregnancy subsequent to failed implantation	Live birth >24/40 at pregnancy subsequent to failed implantation	8	38	–	Chao1, Inverse SImpson	NR	6
Chang^28^	South Korea	S	Vagina	Miscarriage	Term pregnancy	4	55	–	Chao1, Shannon	NR	6
Chen^29^	China	S	Vagina	Miscarriage (5–12/40)	Ongoing pregnancy >12/40	48	116	–	Chao1, Observed species, PD Whole Tree, Shannon	PCoA: Binary Jaccard, Unweighted UniFrac	5
Fan^30^	China	R	Vagina	History of RPL (2+), currently pregnant	Healthy controls, no history of miscarriage, undergoing elective TOP	31	27	–	ACE	PCoA: Unweighted UniFrac	3
Fernández^31^	Spain	R	Vagina	History of RPL (3 + < 12/40)	Healthy controls with 2+ term pregnancies	21	14	–	Shannon, Simpson	PCoA, PERMANOVA: Bray–Curtis	2
Goncharov^32^	Russia	S	Vagina	Miscarriage (5–14/40)	Normal pregnancy	23	23	–	Shannon	PCoA, PERMANOVA: Binary Jaccard	2
Grewal^33^	UK	S (R for subset)	Vagina	First trimester miscarriage	Term pregnancy (after threatened miscarriage)	54 (euploid miscarriage), 39 (aneuploid miscarriage)	74 (70)	–	Inverse Simpson, Observed species	NR	6
Gryaznova^34^	Russia	S	(1) Cervix (2) Vagina	Miscarriage (8–11/40)	Term delivery (with or without dydrogesterone)	11	23 (without dydrogesterone) 17 (with dydrogesterone)	–	Shannon	NMDS, PCoA: Bray–Curtis	5
Gryaznova^55^	Russia	S	Cervix	1st trimester miscarriage	Term delivery (37–42/40)	15	15	–	Shannon	Bray–Curtis	5
Guang^35^	China	S	(1) Cervix (2) Vagina	Missed miscarriage (<12/40)	Elective TOP ( < 12/40)	63	24	Age, gravidity, parity, BMI	Shannon	PCoA: Weighted UniFrac	8
Han^60^	China	S	Endometrium	Spontaneous miscarriage (35–60 days gestation)	(1) Elective TOP (35–60 days) (2) Non-pregnant women undergoing hysteroscopy	56	(1) 39 (2) 10	–	ACE, Chao, Shannon, SImpson	PCoA: Unweighted UniFrac	4
Jiao^36^	China	R	Vagina	History of RPL (2+ consecutive)	Healthy controls, no history of RPL	16	20	–	ACE, Chao, Shannon, SImpson	PCoA, PLS-DA: Bray–Curtis	4
Liu^37^	China	S	Vagina	Miscarriage (<12/40) divided into (1) Empty sac miscarriage defined as empty gestational sac >25 mm (2) Missed miscarriage defined as CRL > 7 mm with absent cardiac activity	Elective TOP (7–12/40)	(1) 13 (2) 22	15	–	Chao1, Shannon	PCoA: Unweighted UniFrac	3
Liu^38^	China	R	(1) Cervix (2) Vagina (3) Endometrium (tissue) (4) Endometrial (lavage)	History of RPL (2+ consecutive)	No history of RPL	(1) 25 (2) 25 (3) 25 (23) (4) 25 (24)	(1) 25 (2) 25 (23) (3) 25 (24) (4) 25 (23)	–	Chao1, Shannon	PCoA, PERMANOVA: Bray–Curtis	4
Liu^61^	China	S	Endometrium	Miscarriage (<10/40) undergoing surgical management	Elective surgical termination of normal pregnancy <10/40	11 (7)	19 (10)	–	Chao1, Shannon	PCA, ANOSIM: Weighted UniFrac	4
Masucci^39^	Italy	R	(1) Vagina (2) Endometrium	History of RPL (2+ consecutive) divided into (1) Genetic susceptibility for coeliac disease (HLA-DQ2/DQ8 positive) and (2) No genetic suspectibility for coeliac disease (HLA-DQ2/DQ8 negative)	Healthy control with previous uncomplicated term pregnancy	(1) 25 (2) 15	7	–	Pielou’s evenness, Shannon	PCoA: Weighted UniFrac	4
McClelland^40^	Kenya	S	Vagina	First-trimester miscarriage	Continuing pregnancy >20/40	(1) Periconception: 45 (2) First trimester: 40	(1) Periconception: 144 (2) First trimester: 140	–	Chao1, Shannon	PCA	9
Moreno^62^	Spain	S	Endometrium	Miscarriage after IVF	Live birth after IVF	3	7	–	NR	NR	4
Moreno^41^	Spain	S	(1)Endometrium (2)Vagina	Spontaneous miscarriage after IVF	Ongoing pregnancy or live birth after IVF	6 (5)	14 (12)	–	Shannon $ Simpson	NR	4
Moreno^63^	International: Europe, Asia, USA	S	Endometrium	(1) Biochemical pregnancy after IVF (2) Clinical miscarriage after IVF	Live birth after IVF	(1) 27 (2) 28	141	–	NR	NR	6
Mori^42^	Japan	R	(1) Cervix (2) Vagina	(Comparison A) History of RPL (2+) (Comparison B) History of RPL (2+), subsequent conception and miscarriage	(A) Healthy non-pregnant controls (B) History of RPL (2+), subsequent conception and live birth	(A) 87 (B) 13 (5 aneuploid, 8 euploid)	(A) 20 (17) (B) 61	–	NR	NR	4
Peuranpaa^43^	Finland	R	(1) Vagina (2) Endometrium	History of RPL (3+ consecutive 1st trimester or 2 with 1+ 2nd trimester)	Healthy controls, no history RPL, investigated for male factor infertility prior to IVF/ICSI	47 (46)	39	Models adjusted for age, parity, BMI	NR	PERMANOVA	7
Seo^56^	South Korea	S	Cervix	History of spontaneous miscarriage (not pregnant)	No history of miscarriage	23	36	–	Chao1, Shannon	NR	5
Severgnini^44^	Italy	S	Vagina	First trimester miscarriage (9–13/40)	Normal pregnancy	9	63	–	Chao1, Good’s coverage, Observed species, PD whole tree, Shannon	PCoA: Weighted and unweighted UniFrac	6
Shahid^45^	Australia	S	Vagina	First trimester miscarriage (<14/40)	Term birth	8	14	–	‘Richness’ ‘Evenness’ Shannon	nMDS, PERMDISP: Bray–Curtis	8
Shi^64^	Japan	R	Endometrium	History of RPL (2+) with subsequent euploid miscarriage	History of RPL (2+) with subsequent live birth	8	30	–	Shannon	NR	7
Shu^65^	China	S	Endometrium	Missed miscarriage (6–8/40)	Elective TOP (6–8/40)	17	12	–	Observed species, Shannon, Chao, Simpson, ACE, Good’s coverage	nMDS: Weighted and Unweighted	3
Skafte-Holm^46^ (Pre-print)	Denmark	S	Vagina	Spontaneous miscarriage <22/40	Term birth	173	1811	–	Inverse Simpson, Shannon	PCoA, PERMANOVA: Metric not reported	6
Sun^47^	China	S	Vagina	Missed miscarriage (<12/40)	Elective TOP (of similar gestational age to cases)	54	50	–	ACE, Chao1, Observed species, Shannon, Simpson	PCoA: Weighted and Unweighted UniFrac, Bray–Curtis	4
Takimoto^66^	Japan	R	Endometrium	History of RPL (2+)	Healthy controls, no history of RPL, at least one normal delivery	27	29	–	NR	NR	3
Tan^48^	China	R	Vagina	(1) History of RPL (2+) consecutive chromosomally normal (2) Clinical miscarriage with history of RPL	(1) Healthy control with at least one live birth (2) Ongoing pregnancy with history of RPL	(1) 34 (2) 11	(1) 15 (2) 13	–	Chao1, Shannon, Simpson	NMDS, PCoA, PERMANOVA: Binary Jaccard, Bray–Curtis, Euclidean distances	5
Van den Tweel^49^	Netherlands	S	Vagina	Miscarriage <12/40 (including biochemical and clinical miscarriage)	Ongoing pregnancy at 12/40	12	17		Shannon	NR	5
Vaughn^67^	Spain	R	Endometrium	History of RPL (3+ or 2+ with one euploid loss)	Healthy control with 1+ live birth and not more than 1 miscarriage	20	10	–	NR	NR	3
Vomstein^68^	Austria	R	Endometrium	History of RPL (3+ consecutive <20/40)	Healthy controls, never pregnant	20	10	–	Chao1, Shannon	PCoA, MDS, PERMANOVA: Bray–Curtis, Weighted and Unweighted UniFrac	4
Wang^50^	China	S	(1) Vagina (2) Endometrium	Missed miscarriage (Intrauterine gestational sac on USS) (7–10/40)	Elective TOP (7–10/40)	38	18	–	Chao1, PD whole tree	PCoA: Bray–Curtis, Unweighted UniFrac	4
Wang^51^	China	Mixed S/R	Vagina	At least one previous miscarriage <12/40	Healthy controls with ≥1 previous live birth	73	105	–	Inverse Simpson, Shannon, Simpson	PCoA, PERMANOVA: Bray–Curtis	4
Wei^69^	China	S	Endometrium	Miscarriage <12/40	Clinical pregnancy (intrauterine pregnancy up to 12/40)	39	194	–	NR	PCoA: Bray–Curtis	7
Xu^52^	China	S	Vagina	Missed miscarriage (confirmed on USS) (<12/40)	Normal early pregnancy	25	25	–	Chao1, Shannon, Simpson	NMDS, PCoA, PCA	3
Zhang^53^	China	R	Vagina	History of RPL (3+ consecutive)	Healthy controls	10	10	-	ACE, Chao, Good’s coverage, Shannon, Simpson ‘Richness’	PCoA Bray–Curtis, Weighted UniFrac	3
Zhao^54^	China	R	Vagina	History of RPL (2+) divided into (1) No medication (2) Drug treatment inc metformin, aspirin	Health controls with history of live birth and no miscarriages	(1) 65 (2) 43 (further subdivisions not reported here)	20 (18)	–	Chao, Shannon	PCoA, MANOVA: Weighted and Unweighted UniFrac	4

*x/40* Weeks of pregnancy, *BMI* Body Mass Index, *ICSI* Intracytoplasmic sperm injection, *IVF* In vitro fertilisation, *MANOVA* Multivariate analysis of variance, *MDS* Multidimensional scaling, *NMDS* Non-metric multidimensional scaling, *NR* Not reported, *PCA* Principal Component Analysis, *PCoA* Principal Coordinate Analysis, *PERMANOVA* Permutational multivariate analysis of variance, *PD Whole tree* Phylogenetic diversity whole tree, *R* Recurrent, *RPL* Recurrent Pregnancy Loss, *S* Spontaneous, *TOP* Termination of pregnancy, *USS* Ultrasound scan

**Fig. 2:**
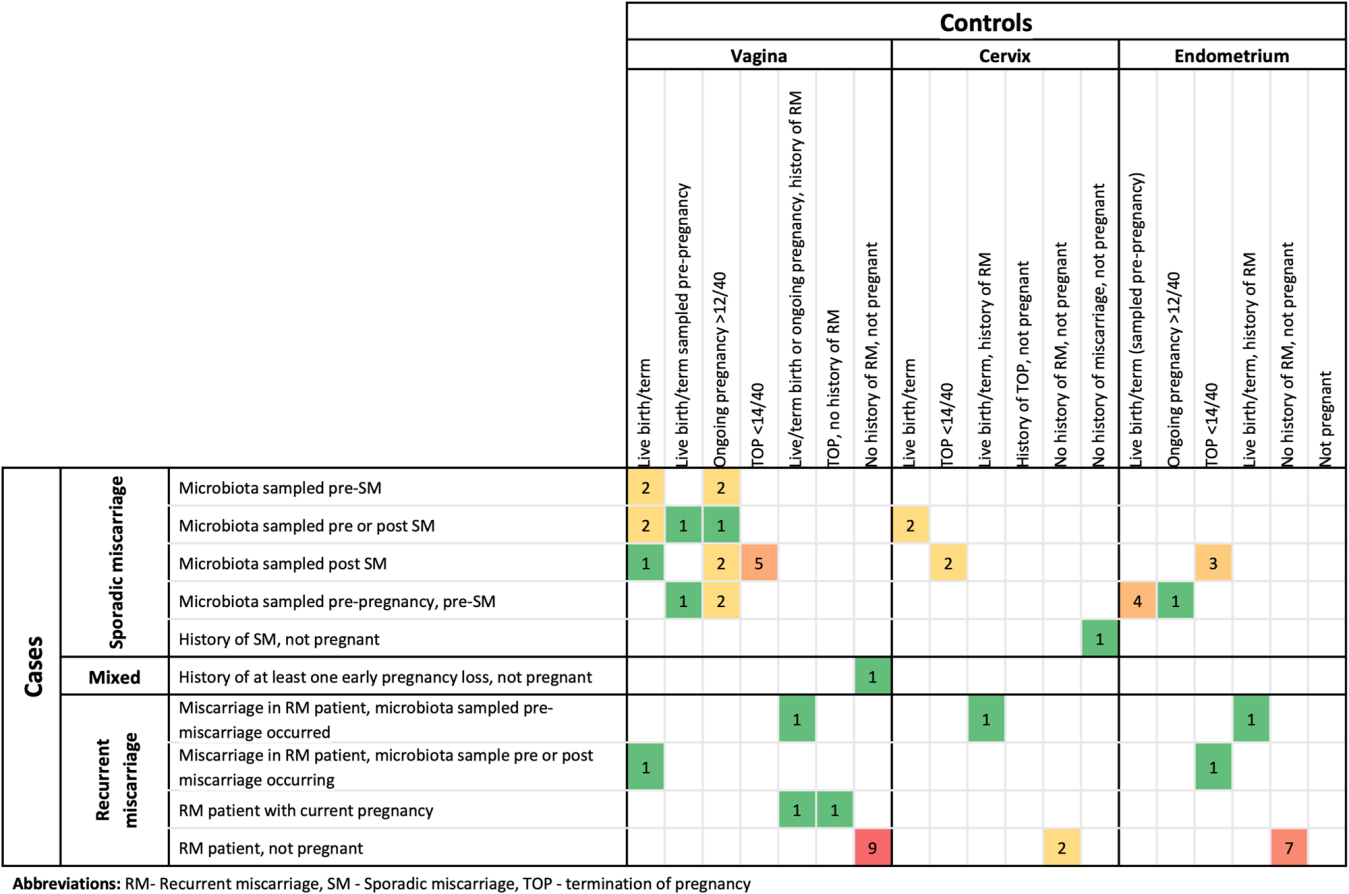
Summary of comparison groups available for included studies.

The case and control definitions varied, as did the sampling times, with some samples taken before and others after the miscarriage had been diagnosed, limiting direct quantitative comparisons. A summary of the comparison groups is presented in Fig. 2. The most common comparison was non-pregnant participants with and without a history of RM, for which the vaginal microbiota was examined by nine studies^31,36,38,39,42,43,48,53,54^ and the endometrial microbiota in seven studies^38,39,43,58,66–68^. For comparison purposes a control group of live/term birth, ongoing pregnancy and termination of otherwise normal pregnancy were combined.

### Microbiota sampling and sequencing methods

A detailed description of the microbiota sampling and sequencing methods is provided in Supplementary Table 4. Sampling was performed by a clinician in all studies except two, where the swabs were self-performed^40,46^. Vaginal and cervical samples were collected using a swab or brush. Endometrial sampling included sheathed and unsheathed sampling devices of endometrial cavity aspirates, endometrial lavage or endometrial tissue. Most samples were frozen at −80 °C immediately (*n* = 27), samples from five studies were chilled and then frozen at −80 °C between four hours and two weeks post collection^31,34,43,55,63^, one study stored samples at −70 °C^40^, one study did not report the frozen temperature^49^, three studies stored samples at 4–8 °C^30,45,58^ and six studies did not report storage methods^32,62,64,66,67,69^.

The method of DNA extraction was reported in 38 of 43 studies. 41 of the 43 studies used 16S ribosomal RNA (rRNA) amplicon sequencing, one study used shotgun metagenomic sequencing^51^ and one study used 2bRad-M sequencing^48^. The hypervariable regions targeted by 16S rRNA sequencing ranged from V1 to V9, with V3–V4 being the most common (18/41). One study also sequenced fungal internal transcribed spacer 1 (Fungal ITS-1)^43^. Sequencing platforms were reported by 39 of 43 studies, including Illumina MiSeq (27/43) Illumina NovaSeq (3/43), Illumina HiSeq (2/43), Roche 454 (3/43), Ion Torrent (1/43), Ion Torrent PGM (1/43), Ion SS XL (1/43) and DNBSEQ Platform (1/43). Negative and/or positive controls were used in 15 studies (15/43, 35%). Adherence to the Strengthening The Organizing and Reporting of Microbiome Studies (STORMS) checklist^70^ ranged from 45% to 88% (Supplementary Tables 4, 5). Areas of poor checklist compliance in the methods included stating the start and end dates for recruitment, follow-up and data collection, lack of sample size justification, unclear durations between sample collection and storage, including time to ship to the laboratory and detailing any methods to control for or identify contamination or confounding by batch effect. Reporting on missing data, generalisability, and providing supplementary data files with all taxa and variable outcomes analysed was also weak. A detailed overview of the sampling and sequencing methods used for each study is presented in Supplementary Table 4.

### Quality assessment

The quality scores according to the Newcastle Ottawa Scale (NOS) ranged from 2 to 9, with a median score of 4. Most studies (69.8%, 30/43) were low quality, followed by moderate quality (20.9%, 9/43)^28,33,43,44,46,59,63,64,69^ and high quality (9.3%, 4/43)^27,35,40,45^. These scores reflect concerns over the selection and representativeness of cases and controls, poor recognition and control for confounding factors and inadequate management or reporting of non-respondents. Individual NOS ratings are provided in the Supplementary Tables 2, 3.

### Assessment of alterations in FRT microbiota

**Fig. 3:**
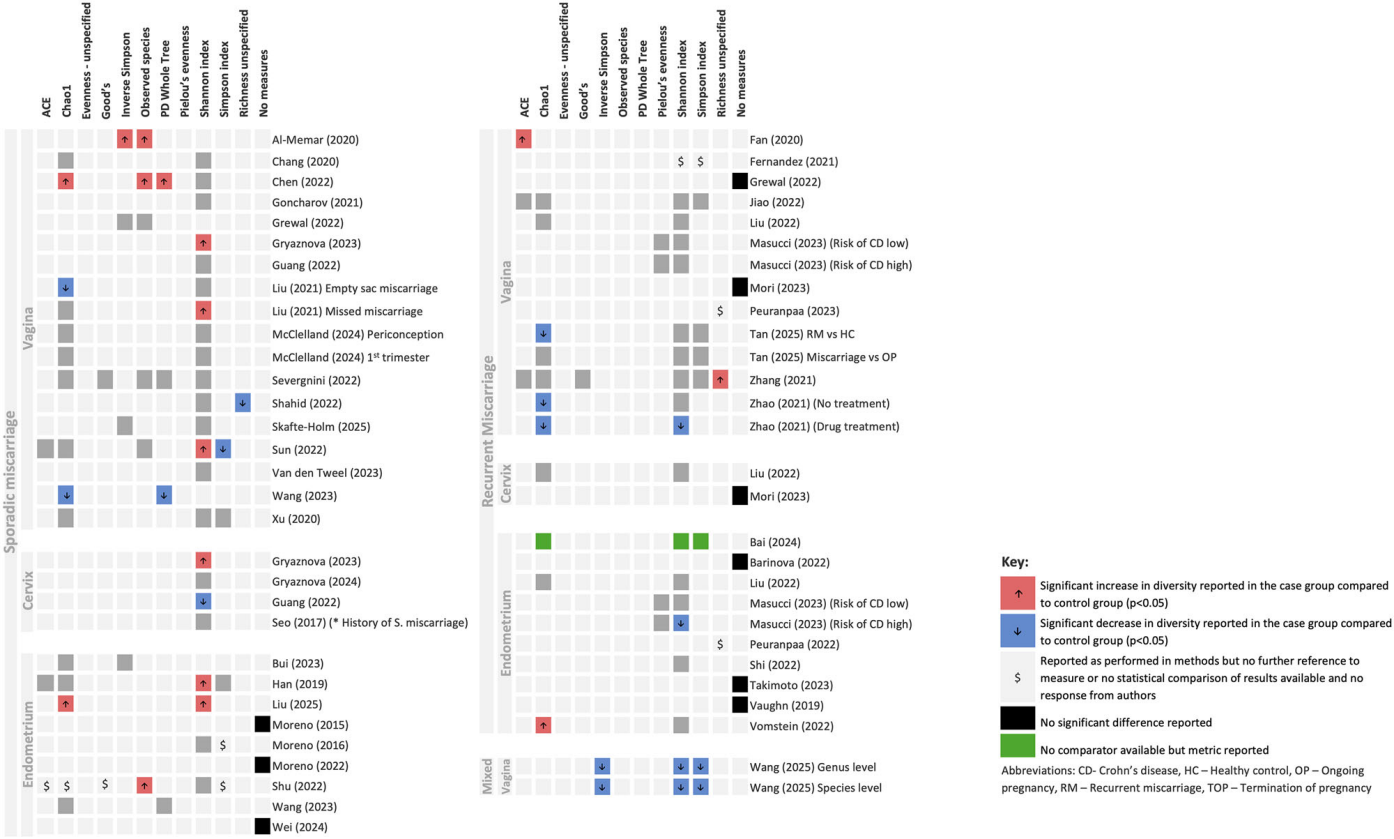
Summary of alpha diversity measures.

The included studies reported a mix of alpha and beta diversity metrics, individual taxa abundance, and taxa changes. α-diversity was assessed in 34 studies using nine indices including richness/evenness (Shannon index, Simpson index, Inverse Simpson index), richness (Abundance Coverage estimator (ACE), Chao1, Observed species), evenness (Pielou), biodiversity (Faith phylogenetic diversity) and sample completeness (Good’s coverage). The most used α-diversity metric was the Shannon index. A summary of the α-diversity measures is presented in Fig. 3.

**Fig. 4:**
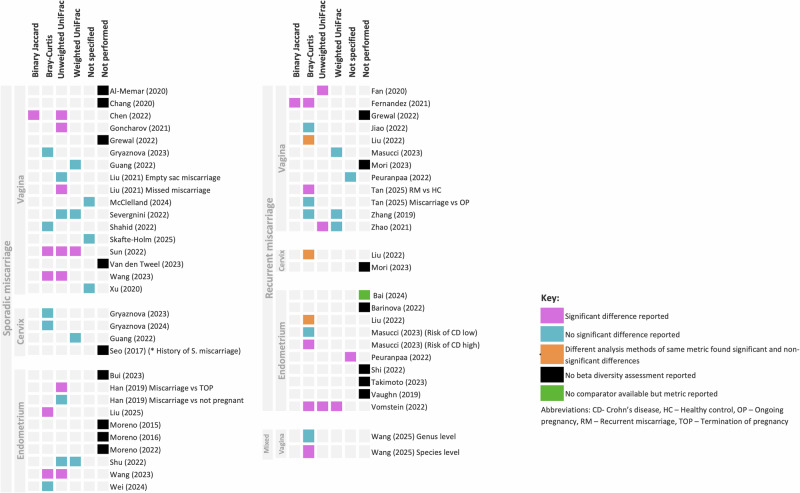
Beta diversity comparison between patients with miscarriage and controls.

ß-diversity was reported by 27 studies (27/43, 62.8%) (Supplementary Table 10). Metrics included Binary Jaccard index, Bray–Curtis dissimilarity and weighted and unweighted UniFrac distances. The most common ß-diversity metric was Bray–Curtis dissimilarity. An overview of the ß-diversity metrics used and whether the studies reported a significant difference between groups is presented in Fig. 4.

The reporting completeness of individual microbial changes at different taxonomic levels varied substantially. Most studies reported changes at a genus level compared to other taxonomic levels. A summary is provided in the Supplementary Material 13–18. Most studies compared diversity and relative abundance of taxa and miscarriage using tests of crude data (Supplementary Tables 13–18).

**Fig. 5:**
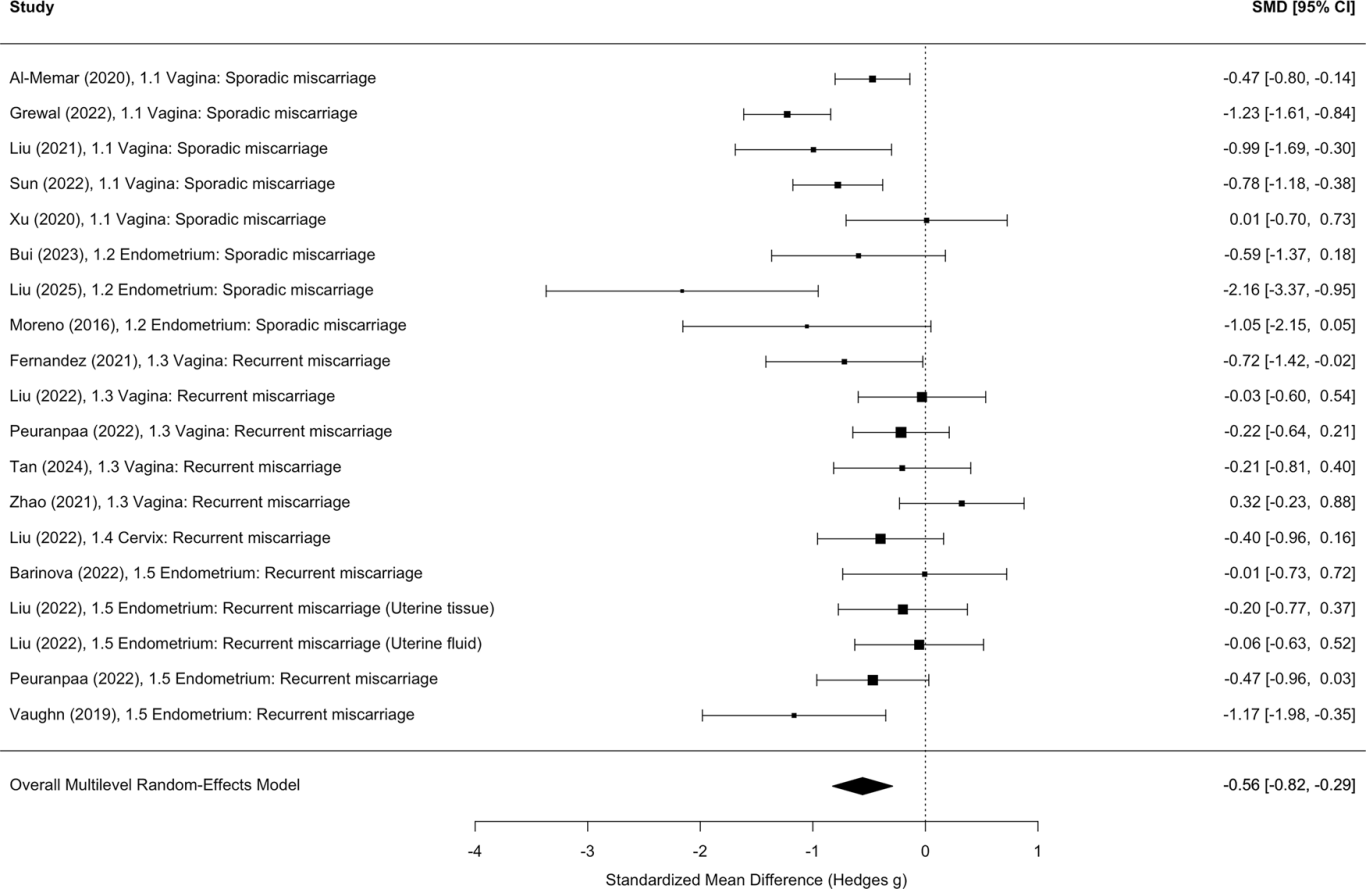
Forest plot of standardised mean difference of relative abundance of *Lactobacillus*.

A multilevel random-effects meta-analysis of *Lactobacillus* abundance across all sample sites in sporadic and recurrent miscarriage cases was reduced across the reproductive tract in miscarriage cases compared to controls (SMD −0.56[95% CI −0.82; −0.29], *p* < 0.01) (Fig. 5). There was substantial heterogeneity among studies, Q(19) = 49.6, *p* < 0.001. The estimated between study variance was s^2^ = 0.18, whereas the within study (subgroup level) variance was effectively zero, indicating that nearly all heterogeneity occurred at the study level (Supplementary Material 6b). Insufficient results for studies rated as high quality were available to conduct a post-hoc sensitivity analysis. However, a reduction in *Lactobacillus* abundance was reported by two high quality studies^27,35^. A post-hoc sensitivity analysis including only studies who reported on 70% of the STORMS checklist also demonstrated a reduction in *Lactobacillus* (SMD −0.59 [95% CI −1.04, −0.15], *p* = 0.008. Significant heterogeneity present (Q(11) = 37.89, *p* < 0.001), driven by difference between studies s^2^ = 0.31, with little to no variation within study subgroups). (Supplementary Material 6a, b, 9b).

### Sporadic miscarriage

Twenty-six studies investigated the association between the FRTM and sporadic miscarriage, sampling the vagina (*n* = 16)^27–29,32–35,37,40,44–47,49,50,52^, cervix (*n* = 4)^34,35,55,56^ and endometrium (*n* = 9)^41,50,59–63,65,69^. There was significant variability in the timing of samples relative to the miscarriage, including sampling pre-pregnancy, pre-miscarriage, post-miscarriage and in those with a history of sporadic miscarriage (Fig. 2).

Shannon index was the most common measure of α-diversity, reported in 81.3% (13/16) of vaginal samples, with three studies reporting a significant increase (23%, 3/13)^34,37,47^. The Shannon index was also tested in all cervical samples, with two studies reporting a significant difference, although with differing directions of association^34,35^. The Shannon index was also the commonest diversity measure in endometrial samples (44.4%, 4/9), with two studies reporting a significant increase^60,61^. Where sufficiently comparable data were available, a multilevel random-effects, meta-analysis of data from eleven differentstudies showed a marginally significant increase in Shannon diversity of patients with sporadic miscarriage (SMD = 0.22, 95% CI [0.05, 0.39], *p* = 0.010) with substantial heterogeneity (Q(12) = 41.29, *p* < 0.001, driven primarily by between study differences) (Supplementary Figs. 7, 9). However, the largest eligible study could not be meta-analysed due to unextractable data but reported no difference in Shannon diversity^46^. No significant differences were found across vaginal and endometrial samples reporting Chao1 diversity (Multilevel model (SMD 0.44, 95% CI [−0.40, 1.28], *p* = 0.30), with substantial heterogeneity (Q(10) = 54.75, *p* < 0.001, driven mainly by between study variation) or Observed species (Standard model, SMD −0.19, 95% CI [−0.86, 0.47], *p* = 0.57, I^2^ = 86.7%,(Supplementary Fig. 7). No strong evidence of publication bias was found but this should be interpreted cautiously (Supplementary Fig. 9).

Significant differences were reported for some ß-diversity metrics but overall, there was no consistency across the different reported metrics and sampling sites (Fig. 4, Supplementary Table 10).

Standard random effects meta-analysis of vaginal samples from sporadic miscarriages revealed a decrease in *Lactobacillus* relative abundance (SMD −0.72, 95% CI [−1.11, −0.34], *p* = 0.003, I^2^ = 71%) compared to healthy controls (Supplementary Material 6c). *Lactobacillus* abundance was also reduced for sporadic miscarriage in the endometrial samples (SMD −1.16, 95% CI [−2.04, −0.28], *p* = 0.01, I^2^ = 53.2%). An overview of reported changes to individual taxa is provided in the Supplementary Tables 13–18. *Lactobacillus* was present in all studies. Of the vaginal studies, six reported a significant decrease in abundance in the case group compared to the controls^27,32,35,37,47,50^, four reported no significant difference^29,44,46,49^ and five did not provide a statistical comparison at genus level^28,33,37,45,52^. The only cervical study reporting to genus level also found a significant decrease in *Lactobacillus* abundance^35^. Statistically significant increases in vaginal *Bacteroides*^29,37,50^ and *Streptococcus*^32,47,51^, among cases of sporadic miscarriage compared with healthy controls, were reported summarily by several studies; however, many studies did not report the abundances or details of their analyses. Thirty-four different genera were reported by at least two research groups in the endometrial studies investigating sporadic miscarriage. Only three studies reported a statistical comparison between genera^50,61,63^, with consistency only reported for decreased *Lactobacillus* in two studies^61,63^.

### Recurrent miscarriage

Seventeen studies examined the association between the FRTM and recurrent miscarriage, including sampling the vagina (*n* = 11)^30,31,33,36,38,39,42,43,48,53,54^, cervix (*n* = 2)^38,42^ and endometrium (*n* = 9)^38,39,43,57,58,64,66–68^. Most studies compared non-pregnant cases with a history of RM to non-pregnant controls without a history of RM (Fig. 2).

Seven different α-diversity metrics were reported across the FRT (Fig. 3), with the Shannon index being the most common – reported in 50% (1/2) of cervical studies, 63.6% (7/11) of vaginal studies and 55.6% (5/9) of endometrial studies. Only one study reported a significant decrease in the Shannon index of a subset of vaginal samples^54^ and another study a significant decrease in a subset of endometrial samples^39^. Overall, no consistent pattern was observed across the other metrics. Meta-analysis of available Shannon and Chao1 data revealed no significant differences (Shannon SMD (−0.15, 95% CI [−0.57, 0.26]), (Chao1 SMD (0.03, 95% CI [−0.70, 0.76]) (Supplementary Figs. 8, 9). Some ß-diversity metrics showed significant differences, but these were inconsistent across metrics and sampling sites, except for unweighted UniFrac in vaginal samples^30,54^ (Fig. 4, Supplementary Table 10).

*Lactobacillus* abundance was reduced across all sites in recurrent miscarriage but did not reach significance (Vaginal samples, random effects model SMD −0.14, 95% CI [−0.42, 0.14], *p* = 0.33, I^2^ 21.8%), cervical sample of one study SMD −0.40, 95% CI [−0.96, 0.16], *p* = 0.16) and endometrial samples multilevel meta-analysis SMD −0.38, 95% CI [−0.78, 0.03], *p* = 0.066), heterogeneity low (σ² = 0.038; corresponding √σ² = 0.195), Q-test not significant (Q(4) = 6.11, *p* = 0.19). An overview of reported individual bacterial taxa changes is presented in the Supplementary Tables 13–18. These comparisons are limited by some studies not presenting a statistical comparison of abundances of individual genera. Alongside a trend of decreased *Lactobacillus*, two studies reported an increase in the genera *Atopobium* and *Streptococcus* in vaginal samples^36,53^. Three studies reported a significant decrease in *Gardnerella* in vaginal samples^30,36,53^. Amongst the few cervical samples, two studies reported a statistically significant increase in *Cutibacterium*^38,42^. Few significant differences were reported for the endometrial sample comparison, with no clear pattern observed.

## Discussion

This systematic review examined changes in the microbiota of the FRT related to both sporadic and recurrent miscarriage. The findings indicate a lower abundance of *Lactobacillus* in FRT samples from miscarriage cases compared to controls. This association was stronger for sporadic than recurrent miscarriage. Vaginal samples from sporadic miscarriages showed a marginal but significant increase in Shannon diversity, but other alpha and beta diversity changes were inconsistent. Beyond *Lactobacillus*, disparate alterations in disease-specific microbes were noted, although potential microbes of interest such as *Bacteroides, Streptococcus* and *Atopobium* were identified. Variability in study populations, sampling, storage, processing methods, and analytical approaches was identified and should be considered when interpreting the summary estimates.

*Lactobacillus* species are key markers of vaginal health, preventing pathogen colonisation through production of lactic acid and anti-microbial compounds such as bacteriocins^71–73^. Vaginal *Lactobacillus* depletion is associated with an increased risk of infection and a link to adverse obstetric outcomes, including preterm birth^10,11^. Reduced *Lactobacillus* has been reported in endometrial studies, however a major limitation of making inferences from these studies is the lack of understanding of what constitutes a ‘normal’ endometrial microbiota. Large, well-designed studies are needed to determine whether *Lactobacillus* is protective in the uterine environment^74,75^.

Despite biological plausibility, the observational nature of studies linking miscarriage and FRTM limits causal conclusions, particularly when many studies did not sample the FRTM prospectively. Unanswered questions persist on whether the vaginal microbiota is established prior to or following conception and whether *Lactobacillus* depletion occurs prior to or following the occurrence of sporadic miscarriage, although we note reduced *Lactobacillus* was observed in the non-pregnant recurrent miscarriage groups. Our findings indicate that *Lactobacillus* depletion warrants attention as a screening tool for miscarriage risk and as a target for intervention. Potential therapies to restore normal microbiota have focused on oral or vaginal application of antibiotics and/or probiotics^76,77^. While changes to both vaginal and endometrial profiles have been successfully reported, these results have had limited clinical translation^78^. Thanaboonyawat et al. showed a significant reduction in miscarriage rates after intravaginal Lactobacilli supplementation before embryo transfer, but no difference biochemical and clinical pregnancy rates^79^. Similarly, Kyono et al. achieved a 20% transition from non-*Lactobacillus* dominant to *Lactobacillus* dominant microbiota following antibiotic and probiotic treatment in IVF patients, but without affecting pregnancy outcomes^80^. Novel therapies, such as vaginal microbiota transplantation, successfully reported by WrØnding et al. provide an intriguing treatment avenue^81^.

Alterations in non-*Lactobacillus* specific microbes were inconsistently reproduced across studies, leading to uncertainty about disease-specific individual microbe alterations. A potential association was observed, with increased vaginal *Bacteroides* in sporadic miscarriages and increased *Streptococcus* in vaginal samples of sporadic and recurrent miscarriages. While both bacteria are common in the reproductive tract, increases in *Bacteroides*, an obligate anaerobe gut commensal has been previously associated with bacterial vaginosis (BV)^82^ and *Streptococcus* has been linked to BV and vaginitis, with the latter implicated in impairing the vaginal epithelium barrier^83,84^. Another microbe of interest in recurrent miscarriage is *Atopobium*, a facultative anaerobic bacteria also associated with BV^85^. Unexpectedly, three studies found a significant decrease in *Gardnerella* in the vaginal samples of recurrent miscarriage patients. However, one study that sampled the vagina in sporadic miscarriage and another sampling the endometrium in recurrent miscarriage reported significant increases in *Gardnerella*; additionally, many studies did not provide statistical comparisons for this genus. Typically, decreases in *Lactobacillus* and increases in *Gardnerella* are associated with BV^86^. However, recent literature has reported an increase in *Gardnerella* abundance in the third trimester of pregnancy, and vaginal colonisation with *Gardnerella* in pregnancy appeared protective against preterm birth^87,88^ suggesting that the role of *Gardnerella* may be more nuanced.

Measures of alpha and beta diversity were widely reported. This meta-analysis found a small but statistically significant increase in Shannon diversity, a measure of richness and evenness, in vaginal samples of sporadic miscarriage. This, together with the observed reduction in *Lactobacillus* abundance, suggests that the loss of a dominant *Lactobacillus* signature may be accompanied by the emergence of a broader range of microbial taxa. However, this change remains uncertain due to limited studies suitable for meta-analysis. The lack of consistent changes in other specific microbes may explain why no clear pattern for other alpha and beta diversity measures were found.

Significant bias was introduced due to variations in study design and laboratory protocols. Several studies did not specify the timing of sample acquisition relative to the miscarriage or menstrual cycle^30,32,34,36,42,45,52–54,56^. Additionally, the time between sample collection and processing varied, as did storage methods, which ranged from immediate freezing at −80 °C to storage at room temperature. These variations are known to affect the subsequent microbiota detected depending on sample type. For example, higher storage temperatures can lead to increased relative abundances of Firmicutes and Actinobacteria, while freezing samples can lead to reduced levels of Bacteroidetes and Proteobacteria^89^. Variations in DNA extraction kits, sequencing platforms, gene sequencing analysis tool and taxa identification databases may all affect downstream results^75^. Raw sequencing data has been publicly uploaded to the NCBI Sequence Read Archive for 42% (18/43) of the included studies and is available on reasonable request from an additional 33% (14/43) studies. A meta-analysis integrating the raw sequencing data through a standardised pipeline may offer further insights and help address some sources of bias. Standardised guidelines on study design, sample processing and analysis are needed to facilitate direct comparison of results from future studies. Standard definitions of sporadic and recurrent miscarriage should be adhered to.

This comprehensive summary of observational studies of FRTM and miscarriage identified a negative association between *Lactobacillus* abundance and miscarriage, which is a potential target for therapeutic intervention. We identified a lack of reproducibility for other findings. We recommend caution be exercised when interpreting findings reporting on the relationship between FRTM and miscarriage and extend this to metagenomic investigations of the FRT, which may be exploratory. Future studies in this field should use standard definitions and methodology and adhere to reporting guidance.

## Methods

A systematic literature review was performed according to the Preferred Reporting Items for Systematic Reviews and Meta-analyses (PRISMA) (Supplementary Table 12). The protocol was prospectively registered (PROSPERO CRD42023415138).

### Search details

MEDLINE, Embase, Cochrane Library, Web of Science, Scopus were searched from inception until 10/06/2025. MeSH terms and keywords included microbiota, microbiome, miscarriage, pregnancy loss, spontaneous abortion and recurrent miscarriage. The full search strategy is in Supplementary Table 1. References and citations of the included articles were manually reviewed for additional articles.

### Selection criteria

Two investigators (NB and IH) independently screened the titles and abstracts, then reviewed full texts for eligible articles using Covidence^90^. A third reviewed (JO) resolved disagreements. The inclusion criteria were the application of an observational design (e.g. case-control study, cohort study, cross-sectional study) and sampling of the FRTM using next generation sequencing, of participants who had experienced a miscarriage or had a history of recurrent pregnancy loss and included a comparison group of healthy controls. Healthy controls were defined as those without a diagnosis of miscarriage or a history of recurrent pregnancy loss. This review focuses specifically on the microbiota, rather than the broader microbiome.

### Data extraction

Two investigators (NB and IH) independently extracted data using a pre-designed template. The primary outcomes were community-level microbiota composition measures, including alpha (α-) and beta (ß-) diversity, taxonomic findings at phylum and genus level and relative abundance of *Lactobacillus*. α-diversity summarises microbial community richness (i.e. number of taxa) and/or evenness (i.e. how well each taxon is represented) in individual samples^91^. ß-diversity measures inter-sample diversity, assessing the phylogenetic structure of communities in comparison with other samples analysed^91^. Additional data on publication details, participant demographics and methodology were also extracted. Each study was assessed against the Strengthening The Organizing and Reporting of Microbiome Studies (STORMS) checklist, a 17-item guide for complete reporting of microbiome studies published in 2021^70^. One investigator performed the assessment, resolving queries with a second reviewer.

Tabular and graphical data from full-text reports were extracted, cleaned and tabulated. In cases where required information was not directly available, the corresponding author was contacted to request additional data. If no response was received and where possible, graphical data was extracted using WebPlotDigitizer(v4.7)^92^. If required, medians and interquartile ranges were transformed to means and standard deviations using a standardised method^93,94^.

### Quality assessment

Two reviewers (NH and IH) independently assessed the methodological quality of studies using the Newcastle-Ottawa Scale (NOS), resolving disagreements through discussion. The NOS evaluates observational studies for internal validity, reliability, comparability and risk of bias based on three criteria: selection, comparability and outcome^95^. A score of ≤5 indicated low quality, 6–7 moderate quality and 8–9 high quality. Participant age, reproductive history and ethnicity were considered potential confounding variables.

### Data synthesis

Differences in α-diversity, ß-diversity and bacterial abundance at both the phylum and genus levels were summarised narratively. Meta-analyses were performed when at least two comparable studies reported the same alpha diversity metric, using random-effects models to calculate the standardised mean difference (SMD) and 95% confidence intervals (CI). Where several studies contributed more than one effect size, such as reporting multiple anatomical sites, a multilevel random-effects meta-analysis was used to account for dependency. Heterogeneity was assessed using estimated variance at each hierarchical level (s^2^) and the multilevel Q-test for residual heterogeneity. Standard random-effects meta-analyses were used when each study contributed only a single, independent effect size with heterogeneity assessed using the I^2^ statistic. The same approach was applied to compare relative *Lactobacillus* abundance between cases and healthy controls. The results were displayed in a forest plot with 95% CIs. Publication bias was assessed visually with funnel plots and Egger’s test for the standard models. For the multilevel meta-analysis, formal tests for publication bias (e.g. Egger’s test) were not conducted because methods that account for dependence are not widely accepted, but standard funnel plots are presented for descriptive purposes, A post-hoc sensitivity analysis including only studies who reported on ≥70% of the STORMS checklist was also performed. Analyses were performed using R studio (2025.09), the code is available at https://osf.io/9scwe/files/8na5e.

## Supplementary information

Supplementary information

Supplementary material

## Data Availability

No new datasets were generated or analysed during the current study. The data used to support the findings of this study are available from the corresponding author upon request.

## Code availability

This is available from https://osf.io/9scwe/files/8na5e.
