# Supplementary material for: Microbiota composition of the female reproductive tract and miscarriage: a systematic review and meta-analysis
Source: NPJ Biofilms Microbiomes. 2026 Jan 8;12:78. doi: 10.1038/s41522-025-00901-9 (PMC13066111; doi:10.1038/s41522-025-00901-9)
Supplement: Supplementary file 1 — Supplementary information [file 41522_2025_901_MOESM1_ESM.docx]

**Microbiota composition of the female reproductive tract and miscarriage: A systematic review and meta-analysis**

Contents

[**Supplementary table 1:** Search strategy: MEDLINE, EMBASE, Cochrane Library, Scopus, Web of Science. 1](#_Toc214392898)

[**Supplementary table 2.** Quality assessment of the case-control studies using the Newcastle-Ottawa Scale (NOS). 4](#_Toc214392899)

[**Supplementary table 3.** Quality assessment of the cohort studies using the Newcastle-Ottawa Scale (NOS). 5](#_Toc214392900)

[**Supplementary table 4.** Details of sampling technique and sample sequencing of the included studies. 7](#_Toc214392901)

[**Supplementary table 5:** Adherence to STORMS checklist 13](#_Toc214392902)

[**Supplementary material 6:** 6a) Forest plot for *Lactobacillus* relative abundance of only studies reporting on ≥70% STORMS checklist 6b) Summary statistics for pooled *Lactobacillus* studies 6c) Summary statistics for pooled subgroup analysis from multilevel meta-analysis of *Lactobacillus* abundance 17](#_Toc214392903)

[**Supplementary figures 7:** Forest plots of alpha diversity measures 7a: Shannon, 7b: Chao, 7c: Observed species in sporadic miscarriage cases and controls, 7d: Summary statistics for forest plots 18](#_Toc214392904)

[**Supplementary figures 8:** Forest plots of alpha diversity measures (8a: Shannon, 8b: Chao) in recurrent miscarriage 8c: Summary statistics for forest plots 19](#_Toc214392905)

[**Supplementary figures 9:** Funnel plots for meta-analyses (9a: Relative abundance of *Lactobacillus*, 9b: Relative abundance of *Lactobacillus* reporting ≥70% STORMS checklist, 9c: Shannon index in sporadic miscarriage, 9d: Chao in sporadic miscarriage, 9e: Observed species in sporadic miscarriage, 9f: Shannon index in recurrent miscarriage, 9g: Chao in recurrent miscarriage) 20](#_Toc214392906)

[**Supplementary table 10.**  Summary of beta diversity measures 23](#_Toc214392907)

[**Supplementary material 11**: r code 26](#_Toc214392908)

[**Supplementary table 12:** Excluded studies after full text review 27](#_Toc214392909)

[**Supplementary table 13:** PRISMA checklist 31](#_Toc214392910)

[**Supplementary tables 14-19:** Summary of individual microbial changes **(see separate Excel document)** 33](#_Toc214392911)

### **Supplementary table 1:** Search strategy: MEDLINE, EMBASE, Cochrane Library, Scopus, Web of Science.

| MEDLINE (OVID) | **Search strategy** | | **No. citations** |
| --- | --- | --- | --- |
|  | 1 | (Miscarriage OR pregnancy loss OR recurrent miscarriage OR recurrent pregnancy loss OR spontaneous abortion OR habitual abortion or recurrent abortion).mp OR exp Abortion, Spontaneous/ OR exp Abortion, Habitual/ | 54741 |
|  | 2 | (Microbiota OR microbiome OR flora OR microflora OR dysbiosis OR 16S OR ecosystem).mp OR exp Microbiota/ OR exp RNA, Ribosomal, 16S/ OR exp dysbiosis/ | 102404 |
|  | 3 | 1 AND 2 | 368 |

| EMBASE (OVID) | **Search strategy** | | **No. citations** |
| --- | --- | --- | --- |
|  | 1 | (Miscarriage OR pregnancy loss OR recurrent miscarriage OR recurrent pregnancy loss OR spontaneous abortion OR habitual abortion OR recurrent abortion).mp OR exp spontaneous abortion/ OR exp recurrent abortion/ | 71116 |
|  | 2 | (Microbiota OR microbiome OR flora OR microflora OR dysbiosis OR 16S OR ecosystem).mp OR exp microflora/ OR exp microbiome/ OR exp bacterial microbiome/ or exp RNA 16S/ OR exp dysbiosis/ | 487133 |
|  | 3 | 1 AND 2 | 703 |

| Web of Science | **Search strategy** | | **No. citations** |
| --- | --- | --- | --- |
|  | 1 | TS=(Miscarriage OR “pregnancy loss” OR “recurrent miscarriage” OR “recurrent pregnancy loss” OR “spontaneous abortion” OR “habitual abortion” OR “recurrent abortion”) | 35754 |
|  | 2 | Microbiota OR microbiome OR flora OR microflora OR dysbiosis OR 16S OR ecosystem | 767602 |
|  | 3 | 1 AND 2 | 431 |

| COCHRANE Library | **Search Strategy** | | **No. citations** |
| --- | --- | --- | --- |
|  | 1 | (Miscarriage OR “pregnancy loss” OR “Recurrent miscarriage” OR “Recurrent pregnancy loss” OR “spontaneous abortion” OR “habitual abortion” OR “recurrent abortion”):ti,ab,kw | 4303 |
|  | 2 | MeSH descriptor: [Abortion, Spontaneous] explode all trees | 1218 |
|  | 3 | MeSH descriptor: [Abortion, Habitual] explode all trees | 387 |
|  | 4 | #1 OR #2 OR #3 | 4749 |
|  | 5 | (Microbiota OR microbiome OR flora OR microflora OR dysbiosis OR 16S OR ecosystem):ti,ab,kw | 15354 |
|  | 6 | MeSH descriptor: [Microbiota] explode all trees | 1806 |
|  | 7 | MeSH descriptor: [RNA, Ribosomal, 16S] explode all trees | 441 |
|  | 8 | MeSH descriptor: [Dysbiosis] explode all trees | 201 |
|  | 9 | #5 OR #6 OR #7 OR #8 | 15360 |
|  | 10 | #4 AND #9 | 47 |

| Scopus | **Search strategy** | | **No. citation** |
| --- | --- | --- | --- |
|  | 1 | TITLE-ABS-KEY ( miscarriage  OR  "pregnancy loss"  OR  "recurrent miscarriage"  OR  "recurrent pregnancy loss"  OR  "spontaneous abortion"  OR  "habitual abortion"  OR  "recurrent abortion" ) | 67572 |
|  | 2 | TITLE-ABS-KEY ( microbiota  OR  microbiome  OR  flora  OR  microflora  OR  dysbiosis  OR  16s  OR  ecosystem ) | 1071758 |
|  | 3 | 1 AND 2 | 705 |

### **Supplementary table 2.** Quality assessment of the case-control studies using the Newcastle-Ottawa Scale (NOS).

| **Case-control studies** | **Selection** | | | | **Comparability** | **Exposure** | | | **Total** |
| --- | --- | --- | --- | --- | --- | --- | --- | --- | --- |
|  | **Case definition** | **Representativeness of the case** | **Selection**  **of controls** | **Definition of controls** | **Controls for age and additional factor** | **Assessment of exposure** | **Same measurement for case and controls** | **Non-respondents** | **0-9** |
| Al-Memar (2020) | 1 | 0 | 1 | 1 | 2 | 1 | 1 | 1 | 8 |
| Bai (2024) | 1 | 0 | 0 | 1 | 0 | 1 | 1 | 0 | 4 |
| Fernandez (2021) | 0 | 0 | 0 | 0 | 0 | 1 | 1 | 0 | 2 |
| Goncharov (2021) | 0 | 0 | 0 | 0 | 0 | 1 | 1 | 0 | 2 |
| Guang (2022) | 1 | 1 | 1 | 1 | 2 | 1 | 1 | 0 | 8 |
| Han (2019) | 1 | 0 | 0 | 1 | 0 | 1 | 1 | 0 | 4 |
| Jiao (2022) | 1 | 0 | 0 | 1 | 0 | 1 | 1 | 0 | 4 |
| Liu (2022) | 1 | 0 | 0 | 1 | 0 | 1 | 1 | 0 | 4 |
| Liu (2021) | 1 | 0 | 0 | 0 | 0 | 1 | 1 | 0 | 3 |
| Liu (2025) | 1 | 0 | 0 | 1 | 0 | 1 | 1 | 0 | 4 |
| Masucci (2023) | 1 | 0 | 0 | 1 | 0 | 1 | 1 | 0 | 4 |
| Mori (2023) | 1 | 0 | 0 | 0 | 0 | 1 | 1 | 1 | 4 |
| Peuranpaa (2022) | 0 | 0 | 1 | 1 | 2 | 1 | 1 | 1 | 7 |
| Seo (2017) | 0 | 0 | 0 | 1 | 2 | 1 | 1 | 0 | 5 |
| Shu (2022) | 1 | 0 | 0 | 0 | 0 | 1 | 1 | 0 | 3 |
| Sun (2022) | 1 | 0 | 0 | 1 | 0 | 1 | 1 | 0 | 4 |
| Takimoto (2023) | 0 | 0 | 0 | 1 | 0 | 1 | 1 | 0 | 3 |
| Vaughn (2019) | 0 | 0 | 0 | 1 | 0 | 1 | 1 | 0 | 3 |
| Vomstein (2022) | 1 | 0 | 0 | 1 | 0 | 1 | 1 | 0 | 4 |
| Wang (2023) | 1 | 0 | 0 | 1 | 0 | 1 | 1 | 0 | 4 |
| Wang (2025) | 1 | 0 | 0 | 1 | 0 | 1 | 1 | 0 | 4 |
| Xu (2020) | 1 | 0 | 0 | 0 | 0 | 1 | 1 | 0 | 3 |
| Zhang (2019) | 0 | 0 | 0 | 1 | 0 | 1 | 1 | 0 | 3 |
| Zhao (2021) | 0 | 0 | 1 | 1 | 0 | 1 | 1 | 0 | 4 |

### **Supplementary table 3.** Quality assessment of the cohort studies using the Newcastle-Ottawa Scale (NOS).

| **Cohort studies** | **Selection** | | | | **Comparability** | **Outcome** | | | **Total** |
| --- | --- | --- | --- | --- | --- | --- | --- | --- | --- |
|  | **Representativeness of exposed cohort** | **Selection of non-exposed cohort** | **Ascertainment of exposure** | **Outcome of interest absent at study start** | **Controls for age and additional factor** | **Assessment of outcome** | **Adequacy of follow up duration** | **Adequate follow up of controls** | **0-9** |
| Barinova (2022) | 0 | 1 | 1 | 1 | 0 | 0 | 1 | 0 | 4 |
| Bui (2023) | 1 | 1 | 1 | 1 | 0 | 0 | 1 | 1 | 6 |
| Chang (2020) | 1 | 1 | 1 | 1 | 0 | 0 | 1 | 1 | 6 |
| Chen (2022) | 1 | 1 | 1 | 1 | 0 | 0 | 0 | 1 | 5 |
| Fan (2020) | 1 | 0 | 1 | 0 | 0 | 1 | 0 | 0 | 3 |
| Grewal (2022) | 1 | 1 | 1 | 0 | 0 | 1 | 1 | 1 | 6 |
| Gryaznova (2023) | 1 | 1 | 1 | 1 | 0 | 0 | 1 | 0 | 5 |
| Gryaznova (2024) | 1 | 1 | 1 | 0 | 0 | 1 | 1 | 0 | 5 |
| McClelland (2024) | 1 | 1 | 1 | 1 | 2 | 1 | 1 | 1 | 9 |
| Moreno (2015) | 0 | 1 | 1 | 1 | 0 | 0 | 1 | 0 | 4 |
| Moreno (2016) | 0 | 1 | 1 | 0 | 0 | 1 | 1 | 0 | 4 |
| Moreno (2022) | 0 | 1 | 1 | 1 | 0 | 1 | 1 | 1 | 6 |
| Severgnini (2022) | 1 | 1 | 1 | 1 | 0 | 1 | 1 | 0 | 6 |
| Shahid (2022) | 1 | 1 | 1 | 1 | 2 | 1 | 1 | 0 | 8 |
| Shi (2022) | 1 | 1 | 1 | 1 | 0 | 1 | 1 | 1 | 7 |
| Skafte-Holm (2025) | 1 | 1 | 1 | 0 | 0 | 1 | 1 | 1 | 6 |
| Tan (2024) | 1 | 0 | 1 | 1 | 0 | 1 | 1 | 0 | 5 |
| Van den Tweel (2024) | 1 | 1 | 1 | 1 | 0 | 1 | 0 | 0 | 5 |
| Wei (2024) | 1 | 1 | 1 | 1 | 2 | 1 | 0 | 0 | 7 |

| **Supplementary table 4.** Details of sampling technique and sample sequencing of the included studies. | | | | | | | | | | | | |  |
| --- | --- | --- | --- | --- | --- | --- | --- | --- | --- | --- | --- | --- | --- |
| **Author (Year)** | **Sampling site** | **Sample timing (cases)** | **Sample timing (controls)** | **Sample collection** | **Sample storage** | **Negative or positive controls** | **Sequencing technique** | **Hyper-**  **variable**  **region** | **DNA extraction method** | **Sequencing platform** | **Average read count/sample (mean unless stated)** | **Publicly available dataset, reference** | **STORMS checklist % reported** |
| **Al-Memar (2020)** | Vagina | 5-8/40, 8-10/40, 10-14/40, >14/40, pre and post miscarriage | 5-8/40, 8-10/40, 10-14/40, >14/40 | Swab of cervicovaginal fluid from posterior vaginal fornix | -80°C | Negative control | 16S rRNA | V1-V2 | QIAamp DNA Mini kit (Qiagen, Manchester, UK) | Ilumina MiSeq | 3267 | Yes, PRJEB32479 | 84% |
| **Bai (2024)** | Endometrium | Prior to uterine curettage for missed miscarriage in early pregnancy | Prior to uterine curettage for elective TOP in early pregnancy | Double lumen embryo transfer catheter to collect uterine cavity fluid | -80°C | Negative controls | 16S rRNA | V3-5 | QIAamp DNA Microbiome Kit (Qiagen, USA) | Illumina MiqSeq | 80973 (RPL only) | On request | 61% |
| **Barinova (2022)** | Endometrium | Cycle day 22-24 | Cycle day 22-24 | Sheathed embryo transfer catheter, covered during uterine insertion/withdrawal | 4°C | NR | 16S rRNA | V3-V4 | Ribo-Prep kit | Ilumina MiSeq | NR | On request | 67% |
| **Bui (2023)** | Endometrium | LH +5-8 in cycle prior to IVF/ICSI | LH +5-8 in cycle prior to IVF/ICSI | Pipelle biopsy | -80°C | Positive (mock community) and negative (blank) controls | 16S rRNA | V1-V2 | Bead-beating and chemical lysis | Ilumina MiSeq | 2167 (median) | Yes, PRJEB53740 | 83% |
| **Chang (2020)** | Vagina | 16-20/40, pre miscarriage | 16-20/40 | Vaginal swab | -80°C | NR | 16S rRNA | V1-V3 | Mobio Soil Kit | Roche 454 NGS platform | 4021 | Yes,  PRJEB3354 | 52% |
| **Chen (2022)** | Vagina | 5-8/40, pre miscarriage | 5-8/40 | Vaginal swab, bilateral vaginal walls | -80°C | NR | 16S rRNA | V3-V4 | DNeasy PowerSoil Kit (Qiagen, Germany) | Ilumina MiSeq | NR | Yes, PRJNA737055 | 84% |
| **Fan (2020)** | Vagina | Not stated | Not stated (presumed prior to TOP) | Vaginal swab of posterior vault secretions | 4-8°C | NR | 16S rRNA | V3-V4 | Trace Flora DNA Extraction Kit-I (Longsee) | Ilumina MiSeq | NR | Yes | 50% |
| **Fernández (2021)** | Vagina | Within 3 days of ovulation | Within 3 days of ovulation | Cervicovaginal lavage with 10ml sterile normal saline | -80°C then -20°C post DNA extraction | NR | 16S rRNA | V3-V4 | QIAamp® Fast DNA Stool Mini Kit (Qiagen, Germantown, MD) | Ilumina MiSeq | 73,383 (median) | On request | 61% |
| **Goncharov (2021)** | Vagina | Not stated | Not stated | Swab of posterior fornix | NR | NR | 16S rRNA | V3-V4 | Ribo-prep kits (Interlabservis, Moscow) | Ilumina MiSeq | NR | NR | 46% |
| **Grewal (2022)** | Vagina | Prior to surgical management of miscarriage | <14/40 | Swab of posterior fornix | -80°C | Negative controls (exposed to clinic and laboratory environments) | 16S rRNA | V1-V2 | QIAamp DNA Mini kit (Qiagen, Manchester, UK) | Ilumina MiSeq | 27,087 | On request | 77% |
| **Gryaznova (2023)** | (1) Cervix (2) Vagina | Not stated but presumed inferred pre miscarriage | Not stated | (1) Cytobrush of cervical canal (2) Vaginal swab | 4°C for 24h, then -80°C | Positive and negative control | 16S rRNA | V3 | ZymoBIOMICS DNA Miniprep Kit | Ion Torrent PGM platform | NR | Yes, PRJNA886610 | 67% |
| **Gryaznova (2024)** | Cervix | 7-11/40 inferred pre miscarriage | 7-11/40 | Cytobrush of cervix | 4°C for 24h, then -80°C | Negative control (Milli-Q water, sterile cytobrush) | 16S rRNA | V3 | Hi Pure DNA Microbiome kit (Magen, Hangzhou, China) | Ion Torrent PGM platform | NR | Yes, PRJNA886610 | 60% |
| **Guang (2022)** | (1) Cervix (2) Vagina | Prior to surgical management of miscarriage | Prior to surgical TOP | (1) Swab inserted 1cm into cervical canal  (2) Swab of vaginal walls | -80°C | NR | 16S rRNA | V4 | QIAamp® DNA Mini Kit (Qiagen, Hilden, Germany) | Ilumina MiSeq | 761,638 | On request | 71% |
| **Han (2019)** | Endometrium | Prior to surgical management of miscarriage | (1) Prior to surgical TOP (2) At hysteroscopy | Negative pressure suction curettage (Biopsy forceps for hysteroscopy) | -80°C | NR | 16S rRNA | V4 | PowerSoil DNA Isolation Kit (Mo Bio Laboratories, USA) | Ilumina MiSeq | 76,044 | NR | 49% |
| **Jiao (2022)** | Vagina | NR | NR | Swab of vaginal wall secretions | -80°C | NR | 16S rRNA | V3-V4 | QIAamp DNA Mini Kit (Qiagen, Hilden, Germany) | Ilumina MiSeq | 53,188 | NR | 50% |
| **Liu (2021)** | Vagina | After miscarriage diagnosis | 7-11 weeks, prior to TOP | Swab of posterior fornix | -80°C | NR | 16S rRNA | V4 | QIAamp DNA Mini kit (Qiagen, Manchester, UK) (presumed) | Ilumina MiSeq | 99,977 | Yes, PRJNA556783 | 56% |
| **Liu (2022)** | (1) Cervix (2) Vagina (3) Endometrium (tissue) (4) Endometrial (lavage) | LH +5-7 | LH +5-7 | (1) Swab of cervical canal (2) Swab of secretions from upper 1/3 of vagina (3) Sheathed negative pressure catheter, covered during insertion and withdrawal (4) Intracervical sheathed artificial insemination tube with 2ml saline injected and withdrawn | -80°C | Negative controls: Normal saline and transfer medium | 16S rRNA | V3-V4 | OMEGA Soil DNA Kit | Ilumina NovaSeq | 71,592 | Yes, PRJNA774109 PRJNA813906 | 70% |
| **Liu (2025)** | Endometrium (secretions) | Prior to surgical management of miscarriage | Prior to surgical TOP | Sterile dry swab of uterine secretions after cleaning vagina and cervix | -80°C | NR | 16S rRNA | V1-V9 | PowerSoil DNA Isolation Kit (Qiagen) | NR | 6078 | NR | 70% |
| **Masucci (2023)** | (1) Vagina (2) Endometrium | Cycle day 19-24 | Cycle day 19-24 | (1) Vaginal swab (2) Tao brush (sheathed) endometrial sampler | -80°C | NR | 16S rRNA | V3-V4 | QIAamp DNA Mini Kit (Qiagen, Hilden, Germany) | Ilumina MiSeq | (1)93,076 (Median)  (2)92,163 (Median) | On request | 59% |
| **McClelland (2024)** | Vagina | (1) Periconception  (2) 9-12/40 | (1) Periconception  (2) 9-12/40 | Clinician and self-collected vaginal swab – Dacron swabs of lateral vaginal walls | -70°C | Positive (mock community) and negative (air and water) controls | 16S rRNA | V3-V4 | QIAamp BiOstic Bacteremia DNA Kit (Qiagen) | Illumina MiSeq | 34370 (Median) | Yes, PRJNA1114047 | 88% |
| **Moreno (2015)** | Endometrium (fluid) | Prior to embryo transfer | Prior to embryo transfer | Endometrial aspiration | NR | NR | 16S rRNA | V3-V5 | NR | NR | NR | NR | Abstract only |
| **Moreno (2016)** | (1)Endometrium (fluid) | LH 2+7 | LH 2+7 | (1)Aspiration of endometrial fluid using intrauterine catheter and syringe | -80°C | NR | 16S rRNA | V3-V5 | MagNa Pure compact nucleic acid isolation kit I (Roche, Madison, WI) | Roche 454 | NR | Yes, SRP078557 | 74% |
| **Moreno (2022)** | (1) Endometrial fluid (2) Endometrial biopsy | Cycle prior to embryo transfer | Cycle prior to embryo transfer | (1) Endometrial fluid aspirated with embryo transfer catheter (2) Cornier cannula scrapping of endometrium | 4°C for 4h, then -80°C | 2-4 blank samples per sequencing run, positive controls: Pure microbial DNA from E.Coli, negative controls: nuclease-free water | 16S rRNA | V2-4-8, V3-6, V7-9 | QIAamp DNA Blood Mini Kit (Qiagen) | Ion S5 XL Sequencer | (1)89,883  (2)103,539 | Yes, PRJNA691300 | 86% |
| **Mori (2023)** | (1) Cervix (2) Vagina | NR | NR | (1) Cervical canal swab (2) Vaginal swab | -80°C | NR | 16S rRNA | V3-V4 | QIAamp DNA Microbiome Kit (Qiagen) | Ilumina MiSeq | NR | NR | 65% |
| **Peuranpaa (2022)** | (1) Vagina (2) Endometrium | LH +6-8 | Preceeding first IVF/ICSI | (1) Vaginal swab from right and left fornices (2) Pipelle endometrial biopsy | -20°C then -80°C within two weeks | Negative and positive controls used | 16S rRNA  Internal transcribed spacer 1 amplicon | V3-V4 (Bacterial) Fungal ITS-1 region (Primers ITSF and ITS2) (Fungal) | Bead beating method | Ilumina MiSeq | (1)23,950  (2)4500 | Yes, PRJEB48310 | 88% |
| **Seo (2017)** | Cervix | NR | NR | Cervix brush (Rovers Medical Devices) | -80°C | Positive controls | 16S rRNA | V1-V3 | Fast DNA SPIN extraction kits | Roche 454 GS Junior platform | NR | Yes,  PRJEB5760 | 64% |
| **Severgnini (2022)** | Vagina | 9-13 weeks | 9-13 weeks (and continuing throughout pregnancy) | Vaginal swab | -80°C | NR | 16S rRNA | V3-V4 | Versant molecular system (Siemens Healthcare Diagnostics, Tarrytown, NY, USA) | Ilumina MiSeq | NR | Yes, PRJNA766806 | 63% |
| **Shahid (2022)** | Vagina | NR | NR | Decron tip swab of vaginal midpoint | 4°C | Negative controls (duplicates of four reagents from DNA extraction kit) | 16S rRNA | V4 | QIAamp DNA Mini Kit (QIAGEN, Chadstone Centre, Victoria, Australia) | Ilumina MiSeq | NR | Yes, PRJEB47614 | 75% |
| **Shi (2022)** | Endometrium | Midluteal phase | Midluteal phase | Tissue aspirated with sampling pipette (Pipet Curet) | NR | NR | 16S rRNA | V4 | NR | Ilumina MiSeq | NR | NR | 60% |
| **Shu (2022)** | Endometrium (fluid) | Prior to surgical management of miscarriage | Prior to surgical TOP | Sheathed endometrial sampler (similar to Tao brush) | -80°C | NR | 16S rRNA | V3-V4 | SDS extraction | Illumina NovaSeq | 62,048 | On request | 52% |
| **Skafte-Holm (2025)**  **** Pre-print** | Vagina | Enrolment in study 11-14/40 or emergency admission with suspected miscarriage | Enrolment in study 11-14/40 or emergency admission with suspected miscarriage | Self-collected high vaginal swab (flocked swab) | -80°C | NR | 16S rRNA | V3-V4 | Pathogen Universal 200 MagNAPure (Roche Molecular Systems Inc.m CA, USA) | Illumina MiSeq | Case: 10164 (median)  Control: 9229 (median) | On request | 82% |
| **Sun (2022)** | Vagina | Immediately after diagnosis of miscarriage | At decision for elective TOP | Vaginal swab of posterior fornix | -80°C | NR | 16S rRNA | V1-V9 | Microorganism DNA extraction kit (Halgen Lts., Guangzhou, China) | NR | NR | NR | 56% |
| **Takimoto (2023)** | Endometrium | Midluteal phase | Midluteal phase | Aspiration using sampling pipette | NR | NR | 16S rRNA | V4 | NR | Ilumina MiSeq | NR | NR | 46% |
| **Tan (2025)** | Vagina | Midluteal phase | Midluteal phase | Flocked swabs from right and left fornices | -80°C | NR | 2bRAD-M | n/a | NR | Illumina NovaSeq PE150 | 283446 | On request | 60% |
| **Van den Tweel (2023)** | Vagina | Pre-IVF/IUI not menstruating | Pre-IVF/IUI not menstruating | Swab of posterior fornix | Frozen within 24h | Positive (ZymoBIOICS) and negative (Phosphate-Buffered Saline) controls | 16S rRNA | V1-V2 | MagNA Pure 96 (Roche Diagnostics, Basel, Switzerland) | Illumina MiSeq | NR | On request | 79% |
| **Vaughn (2019)** | Endometrium (fluid) | LH +6 | LH +6 | Intrauterine catheter | NR | NR | 16S rRNA | NR | NR | NR | NR | NR | Abstract only |
| **Vomstein (2022)** | Endometrium (fluid) | Follicular phase, midcycle (ovulation), luteal phase | Follicular phase, midcycle (ovulation), luteal phase | Flexible catheter with uterine flushing and suction retrieval | -80°C | Paired vaginal fornix and endometrial samples to examine for potential contamination, negative controls used. | 16S rRNA | V3-V4 | Qiagen DNA Microbiome kit | Ilumina MiSeq | NR | Yes, PRJEB44227 | 75% |
| **Wang (2023)** | (1) Vagina (2) Endometrium | After miscarriage, prior to surgical management of miscarriage | Prior to surgical TOP | (1) Swab of vaginal walls (2) Sheathed endometrial sampling brush | -80°C | NR | 16S rRNA | V4 | ZymoBIOMICS DNA Miniprep Kit | Illumina HiSeq | (1)34,309  (2)34,296 | Yes, PRJNA933192 | 64% |
| **Wang (2025)** | Vagina | After diagnosis of early pregnancy loss during menstrual phase | Menstrual phase | Swab of posterior vaginal fornix | -80°C | NR | Shotgun metagenomic sequencing | n/a | Phenol/Tri-chloromethane method | DNBSEQ Platform (BGI-Shenzhen, Shenzhen, China) | 11.78 Gb | Yes, BioSample SUB14915711 | 69% |
| **Wei (2024)** | Endometrium | At embryo transfer | At embryo transfer | 1cm portion of inner embryo transfer catheter (sheathed during insertion and removal) | NR | Negative (black control and transfer media) control | 16S rRNA | V3-V4 | FastDNA Spin Kit (MP Biomedicals, Santa Ana, CA, USA) | Illumina MiSeq PE300 | NR | On request | 70% |
| **Xu (2020)** | Vagina | After miscarriage diagnosis | NR | Swab of lateral wall of midvagina | -80°C | NR | 16S rRNA | V4 | QIAamp Fast DNA Stool Mini Kit | Illumina HiSeq | NR | NR | 48% |
| **Zhang (2019)** | Vagina | NR | NR | Swab of vaginal wall secretions | -80°C | NR | 16S rRNA | V3-V4 | QIAamp DNA Mini Kit (Qiagen, Hilden, Germany) | Ilumina MiSeq | 37,299 | On request | 48% |
| **Zhao (2021)** | Vagina | NR | NR | Swab of lateral vaginal wall | -80°C | NR | 16S rRNA | V3-V4 | FastDNA® SPIN Kit for Soil (MP Biomedicals, Ohio, USA) | Ilumina MiSeq | 45,706 | Yes, PRJNA683172 | 73% |
|  |  | **Abbreviations:** : x/40 – Weeks of pregnancy, 16S rRNA – 16S ribosomal RNA, DNA – Deoxyribonucleic acid, ITS – Internal Transcribed Spacer, ICSI – Intracytoplasmic sperm injection, IVF – In vitro fertilisation, LH – Luteinising hormone (surge), NR – Not reported, TOP – Termination of Pregnancy, Vx – Variable region | | | | | | | | | | |  |

### **Supplementary table 5:** Adherence to STORMS checklist

| **Reporting by STORMS checklist** | | | | | |
| --- | --- | --- | --- | --- | --- |
| Version: | 1.03 |  | **Reported by studies** | | |
| **Number** | **Item** | **Recommendation** | Y | N | N/A |
| **Abstract** | |  |  |  |  |
| 1.0 | Structured or Unstructured Abstract | Abstract should include information on background, methods, results, and conclusions in structured or unstructured format. | 97.6 | 2.4 | 0.0 |
| 1.1 | Study Design | State study design in abstract. | 39.0 | 61.0 | 0.0 |
| 1.2 | Sequencing methods | State the strategy used for metagenomic classification. | 92.7 | 7.3 | 0.0 |
| 1.3 | Specimens | Describe body site(s) studied. | 100.0 | 0.0 | 0.0 |
| **Introduction** | | |  |  |  |
| 2.0 | Background and Rationale | Summarize the underlying background, scientific evidence, or theory driving the current hypothesis as well as the study objectives. | 95.1 | 4.9 | 0.0 |
| 2.1 | Hypotheses | State the pre-specified hypothesis. If the study is exploratory, state any pre-specified study objectives. | 95.1 | 4.9 | 0.0 |
| **Methods** | |  |  |  |  |
| 3.0 | Study Design | Describe the study design. | 63.4 | 36.6 | 0.0 |
| 3.1 | Participants | State what the population of interest is, and the method by which participants are sampled from that population. Include relevant information on physiological state of the subjects or stage in the life history of disease under study when participants were sampled. | 97.6 | 2.4 | 0.0 |
| 3.2 | Geographic location | State the geographic region(s) where participants were sampled from. | 82.9 | 17.1 | 0.0 |
| 3.3 | Relevant Dates | State the start and end dates for recruitment, follow-up, and data collection. | 12.2 | 87.8 | 0.0 |
| 3.4 | Eligibility criteria | List any criteria for inclusion and exclusion of recruited participants. | 92.7 | 7.3 | 0.0 |
| 3.5 | Antibiotics Usage | List what is known about antibiotics usage before or during sample collection. | 68.3 | 31.7 | 0.0 |
| 3.6 | Analytic sample size | Explain how the final analytic sample size was calculated, including the number of cases and controls if relevant, and reasons for dropout at each stage of the study. This should include the number of individuals in whom microbiome sequencing was attempted and the number in whom microbiome sequencing was successful. | 26.8 | 73.2 | 0.0 |
| 3.7 | Longitudinal Studies | For longitudinal studies, state how many follow-ups were conducted, describe sample size at follow-up by group or condition, and discuss any loss to follow-up. | 27.5 | 35.0 | 37.5 |
| 3.8 | Matching | For matched studies, give matching criteria. | 5.0 | 0.0 | 95.0 |
| 3.9 | Ethics | State the name of the institutional review board that approved the study and protocols, protocol number and date of approval, and procedures for obtaining informed consent from participants. | 82.9 | 17.1 | 0.0 |
| 4.0 | Laboratory methods | State the laboratory/center where laboratory work was done. | 31.7 | 68.3 | 0.0 |
| 4.1 | Specimen collection | State the body site(s) sampled from and how specimens were collected. | 97.6 | 2.4 | 0.0 |
| 4.2 | Shipping | Describe how samples were stored and shipped to the laboratory. | 29.3 | 70.7 | 0.0 |
| 4.3 | Storage | Describe how the laboratory stored samples, including time between collection and storage and any preservation buffers or refrigeration used. | 14.6 | 85.4 | 0.0 |
| 4.4 | DNA extraction | Provide DNA extraction method, including kit and version if relevant. | 80.5 | 19.5 | 0.0 |
| 4.5 | Human DNA sequence depletion or microbial DNA enrichment | Describe whether human DNA sequence depletion or enrichment of microbial or viral DNA was performed. | 0.0 | 0.0 | 100.0 |
| 4.6 | Primer selection | Provide primer selection and DNA amplification methods as well as variable region sequenced (if applicable). | 82.9 | 12.2 | 4.9 |
| 4.7 | Positive Controls | Describe any positive controls (mock communities) if used. | 17.1 | 0.0 | 82.9 |
| 4.8 | Negative Controls | Describe any negative controls if used. | 31.7 | 0.0 | 68.3 |
| 4.9 | Contaminant mitigation and identification | Provide any laboratory or computational methods used to control for or identify microbiome contamination from the environment, reagents, or laboratory. | 34.1 | 65.9 | 0.0 |
| 4.10 | Replication | Describe any biological or technical replicates included in the sequencing, including which steps were replicated between them. | 9.8 | 0.0 | 90.2 |
| 4.11 | Sequencing strategy | Major divisions of strategy, such as shotgun or amplicon sequencing. | 95.1 | 4.9 | 0.0 |
| 4.12 | Sequencing methods | State whether experimental quantification was used (QMP/cell count based, spike-in based) or whether relative abundance methods were applied. | 92.7 | 7.3 | 0.0 |
| 4.13 | Batch effects | Detail any blocking or randomization used in study design to avoid confounding of batches with exposures or outcomes. Discuss any likely sources of batch effects, if known. | 2.4 | 97.6 | 0.0 |
| 5.0 | Data sources/ measurement | For each non-microbiome variable, including the health condition, intervention, or other variable of interest, state how it was defined, how it was measured or collected, and any transformations applied to the variable prior to analysis. | 48.8 | 51.2 | 0.0 |
| 6.0 | Research design for causal inference | Discuss any potential for confounding by variables that may influence both the outcome and exposure of interest. State any variables controlled for and the rationale for controlling for them. | 22.0 | 78.0 | 0.0 |
| 6.1 | Selection bias | Discuss potential for selection or survival bias. | 17.1 | 82.9 | 0.0 |
| 7.0 | Bioinformatic and Statistical Methods | Describe any transformations to quantitative variables used in analyses (e.g. use of percentages instead of counts, normalization, rarefaction, categorization). | 58.5 | 29.3 | 12.2 |
| 7.1 | Quality Control | Describe any methods to identify or filter low quality reads or samples. | 90.2 | 9.8 | 0.0 |
| 7.2 | Sequence analysis | Describe any taxonomic, functional profiling, or other sequence analysis performed. | 95.1 | 4.9 | 0.0 |
| 7.3 | Statistical methods | Describe all statistical methods. | 78.0 | 22.0 | 0.0 |
| 7.4 | Longitudinal analysis | If the study is longitudinal, include a section that explicitly states what analysis methods were used (if any) to account for grouping of measurements by individual or patterns over time. | 19.5 | 17.1 | 63.4 |
| 7.5 | Subgroup analysis | Describe any methods used to examine subgroups and interactions. | 48.8 | 4.9 | 46.3 |
| 7.6 | Missing data | Explain how missing data were addressed. | 22.5 | 77.5 | 0.0 |
| 7.7 | Sensitivity analyses | Describe any sensitivity analyses. | 26.8 | 0.0 | 73.2 |
| 7.8 | Findings | State criteria used to select findings for reporting. | 92.7 | 7.3 | 0.0 |
| 7.9 | Software | Cite all software (including read mapping software) and databases (including any used for taxonomic reference or annotating amplicons, if applicable) used. Include version numbers. | 95.1 | 4.9 | 0.0 |
| 8.0 | Reproducible research | Make a statement about whether and how others can reproduce the reported analysis. | 46.3 | 53.7 | 0.0 |
| 8.1 | Raw data access | State where raw data may be accessed including demultiplexing information. | 46.3 | 53.7 | 0.0 |
| 8.2 | Processed data access | State where processed data may be accessed. | 31.7 | 68.3 | 0.0 |
| 8.3 | Participant data access | State where individual participant data such as demographics and other covariates may be accessed, and how they can be matched to the microbiome data. | 24.4 | 75.6 | 0.0 |
| 8.4 | Source code access | State where code may be accessed. | 4.9 | 22.0 | 73.2 |
| 8.5 | Full results | Provide full results of all analyses, in computer-readable format, in supplementary materials. | 14.6 | 85.4 | 0.0 |
| **Results** | |  |  |  |  |
| 9.0 | Descriptive data | Give characteristics of study participants (e.g. dietary, demographic, clinical, social) and information on exposures and potential confounders. | 87.8 | 12.2 | 0.0 |
| 10.0 | Microbiome data | Report descriptive findings for microbiome analyses with all applicable outcomes and covariates. | 85.4 | 14.6 | 0.0 |
| 10.1 | Taxonomy | Identify taxonomy using standardized taxon classifications that are sufficient to uniquely identify taxa. | 100.0 | 0.0 | 0.0 |
| 10.2 | Differential abundance | Report results of differential abundance analysis by the variable of interest and (if applicable) by time, clearly indicating the direction of change and total number of taxa tested. | 70.7 | 29.3 | 0.0 |
| 10.3 | Other data types | Report other data analyzed--e.g. metabolic function, functional potential, MAG assembly, and RNAseq. | 10.0 | 0.0 | 90.0 |
| 10.4 | Other statistical analysis | Report any statistical data analysis not covered above. | 0.0 | 0.0 | 100.0 |
| **Discussion** | |  |  |  |  |
| 11.0 | Key results | Summarise key results with reference to study objectives | 100.0 | 0.0 | 0.0 |
| 12.0 | Interpretation | Give a cautious overall interpretation of results considering objectives, limitations, multiplicity of analyses, results from similar studies, and other relevant evidence. | 87.8 | 12.2 | 0.0 |
| 13.0 | Limitations | Discuss limitations of the study, taking into account sources of potential bias or imprecision. | 75.6 | 24.4 | 0.0 |
| 13.1 | Bias | Discuss any potential for bias to influence study findings. | 61.0 | 39.0 | 0.0 |
| 13.2 | Generalizability | Discuss the generalisability (external validity) of the study results | 36.6 | 63.4 | 0.0 |
| 14.0 | Ongoing/future work | Describe potential future research or ongoing research based on the study's findings. | 26.8 | 0.0 | 73.2 |
| **Other information** | | |  |  |  |
| 15.0 | Funding | Give the source of funding and the role of the funders for the present study and, if applicable, for the original study on which the present article is based | 95.1 | 4.9 | 0.0 |
| 15.1 | Acknowledgements | Include acknowledgements of those who contributed to the research but did not meet critera for authorship. | 92.7 | 7.3 | 0.0 |
| 15.2 | Conflicts of Interest | Include a conflicts of interest statement. | 100.0 | 0.0 | 0.0 |
| 16.0 | Supplements | Indicate where supplements may be accessed and what materials they contain. | 65.9 | 0.0 | 34.1 |
| 17.0 | Supplementary data | Provide supplementary data files of results with for all taxa and all outcome variables analyzed. Indicate the taxonomic level of all taxa. | 17.1 | 80.5 | 2.4 |

### **Supplementary material 6:** 6a) Forest plot for *Lactobacillus* relative abundance of only studies reporting on ≥70% STORMS checklist 6b) Summary statistics for pooled *Lactobacillus* studies 6c) Summary statistics for pooled subgroup analysis from multilevel meta-analysis of *Lactobacillus* abundance

**Figure 6a: Forest plot for *Lactobacillus* relative abundance ≥70% STORMS checklist**


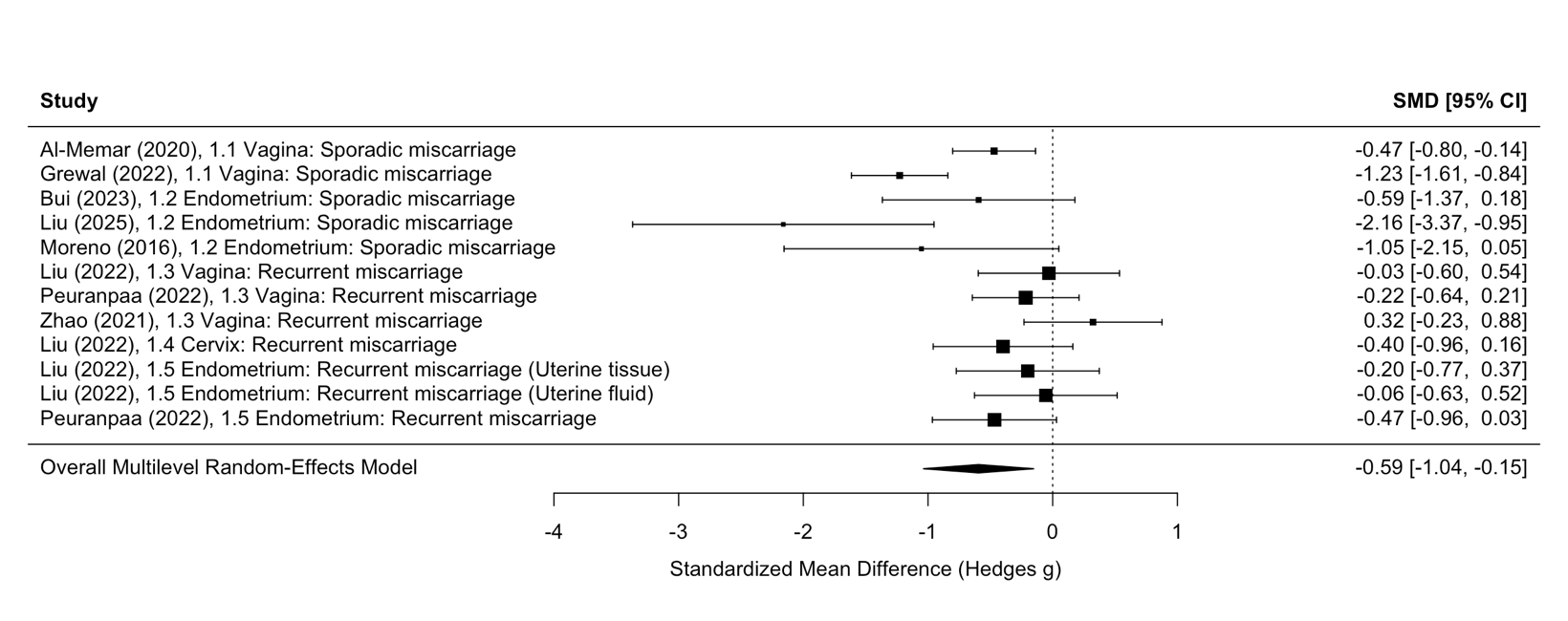


| **6b) Summary statistics for pooled *Lactobacillus* studies** | | | | | | | |
| --- | --- | --- | --- | --- | --- | --- | --- |
| **Model results** | **k** | **SMD Estimate** | **95% CI** | **p-value** | **Q(df), p** | **σ²₁ (Between-study)** | **σ²₂ (Within-study)** |
| **Lactobacillus abundance** | 19 | –0.56 | –0.82 to –0.29 | < .001 | Q(18) = 49.64, p < .001 | 0.178 | 0 |
| **Sensitivity analysis >70% STORMS** | 12 | –0.59 | –1.04 to –0.15 | 0.008 | Q(11) = 37.89, p < .001 | 0.309 | 0 |

| **6c) Summary statistics for pooled subgroup analysis from multilevel meta-analysis of *Lactobacillus* abundance** | | | |
| --- | --- | --- | --- |
| **Subgroup** | **k** | **Pooled SMD (g)** | **95% CI** |
| **1.1 Vagina: Sporadic miscarriage** | 5 | **−0.72** | −1.11 to −0.34 |
| **1.2 Endometrium: Sporadic miscarriage** | 3 | **−1.16** | −2.04 to −0.28 |
| **1.3 Vagina: Recurrent miscarriage** | 5 | **−0.14** | −0.42 to 0.14 |
| **1.4 Cervix: Recurrent miscarriage** | 1 | **−0.40** | −0.96 to 0.16 |
| **1.5 Endometrium: Recurrent miscarriage** | 5 | **−0.38** | −0.78 to 0.03 |

### **Supplementary figures 7:** Forest plots of alpha diversity measures 7a: Shannon, 7b: Chao, 7c: Observed species in sporadic miscarriage cases and controls, 7d: Summary statistics for forest plots

**Figure 7a: Shannon index in sporadic miscarriage**


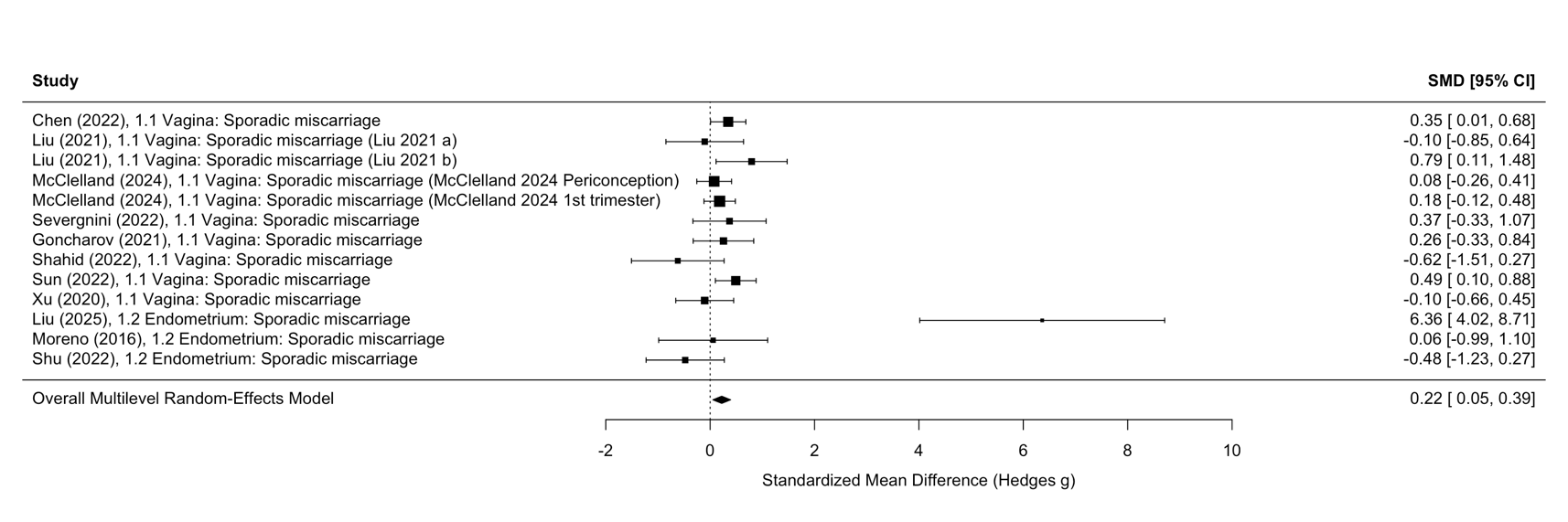


**Figure 7b: Chao1 in sporadic miscarriage**

*
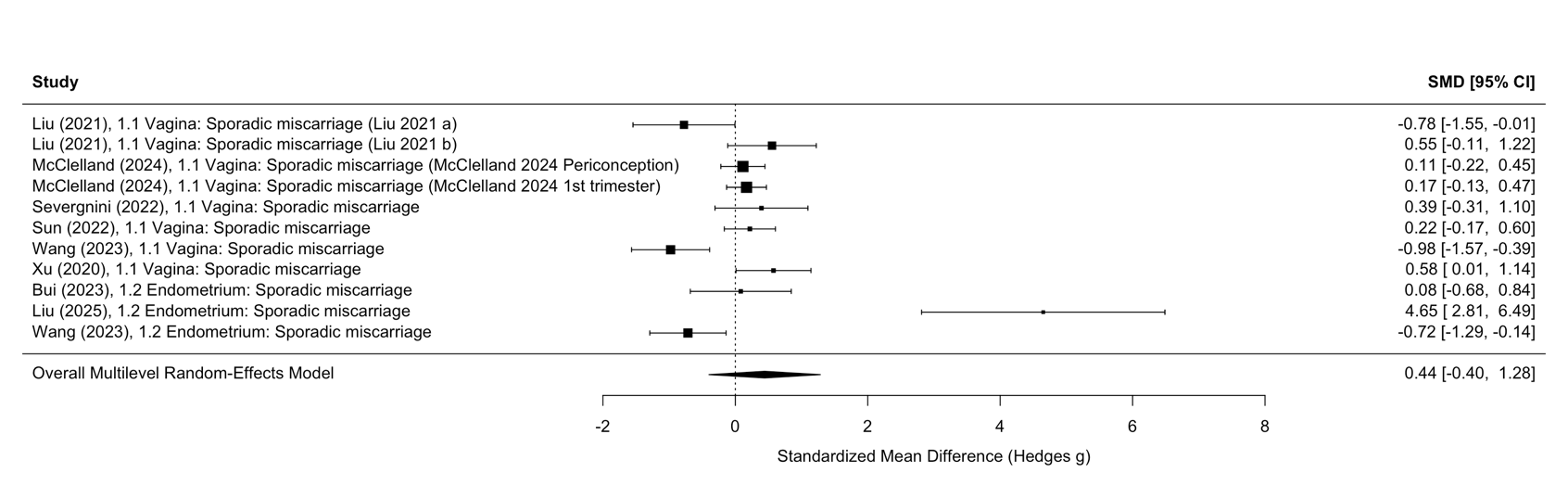
*

**Figure 7c: Observed species in sporadic miscarriage**

*
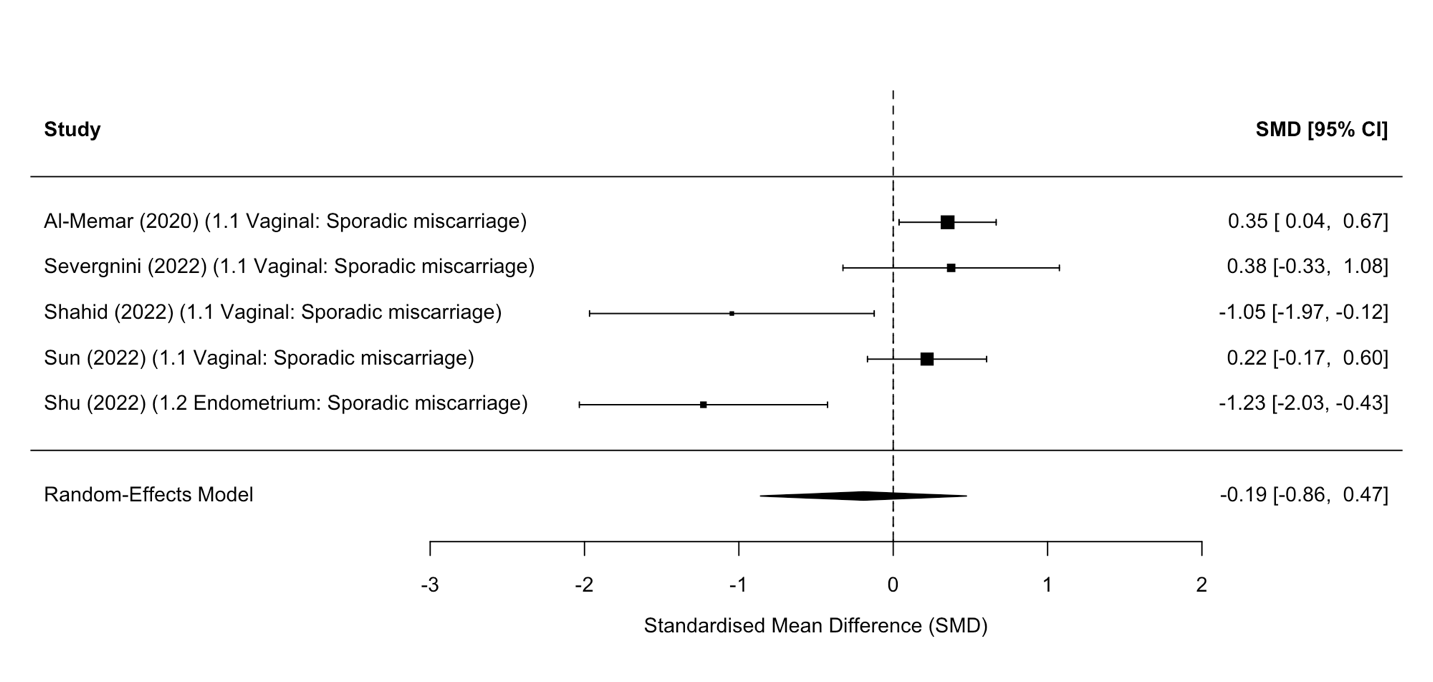
*

| **7d: Summary statistics for forest plots** | | | | | | | |
| --- | --- | --- | --- | --- | --- | --- | --- |
| **Model** | **K** | **SMD Estimate** | **95% CI** | **p-value** | **Q(df), p** | **σ²₁ (Between-study)** | **σ²₂ (Within-study)** |
| **Shannon diversity sporadic (7a)** | 13 | 0.22 | 0.05 to 0.39 | 0.01 | Q(12) = 41.29, p < .001 | 0.0139 | 0 |
| **Chao1 diversity sporadic (7b)** | 11 | 0.44 | –0.40 to 1.28 | 0.3 | Q(10) = 54.75, p < .001 | 1.261 | 0.094 |
| **Observed species sporadic (7c)** | 5 | –0.19 | –0.86 to 0.47 | 0.57 | Q(4) = 19.83, p = .0005 | 0.236 | 0.236 |

### **Supplementary figures 8:** Forest plots of alpha diversity measures (8a: Shannon, 8b: Chao) in recurrent miscarriage 8c: Summary statistics for forest plots

**Figure 8a: Shannon index in recurrent miscarriage**

***
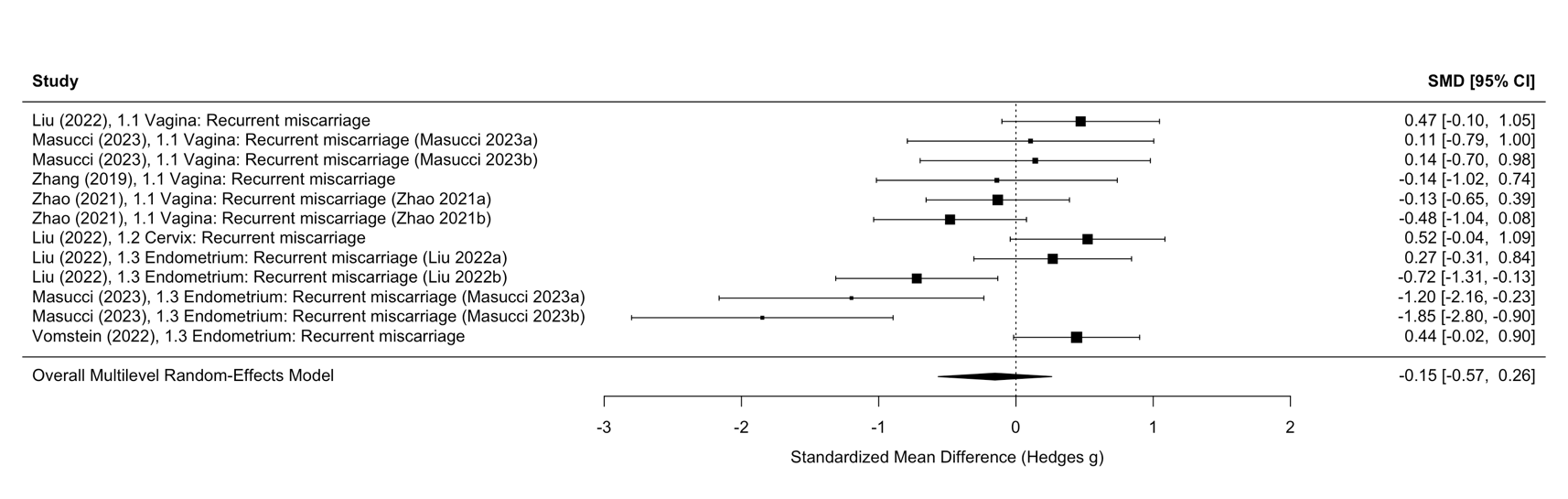
***

**Figure 8b: Chao1 in recurrent miscarriage**

***
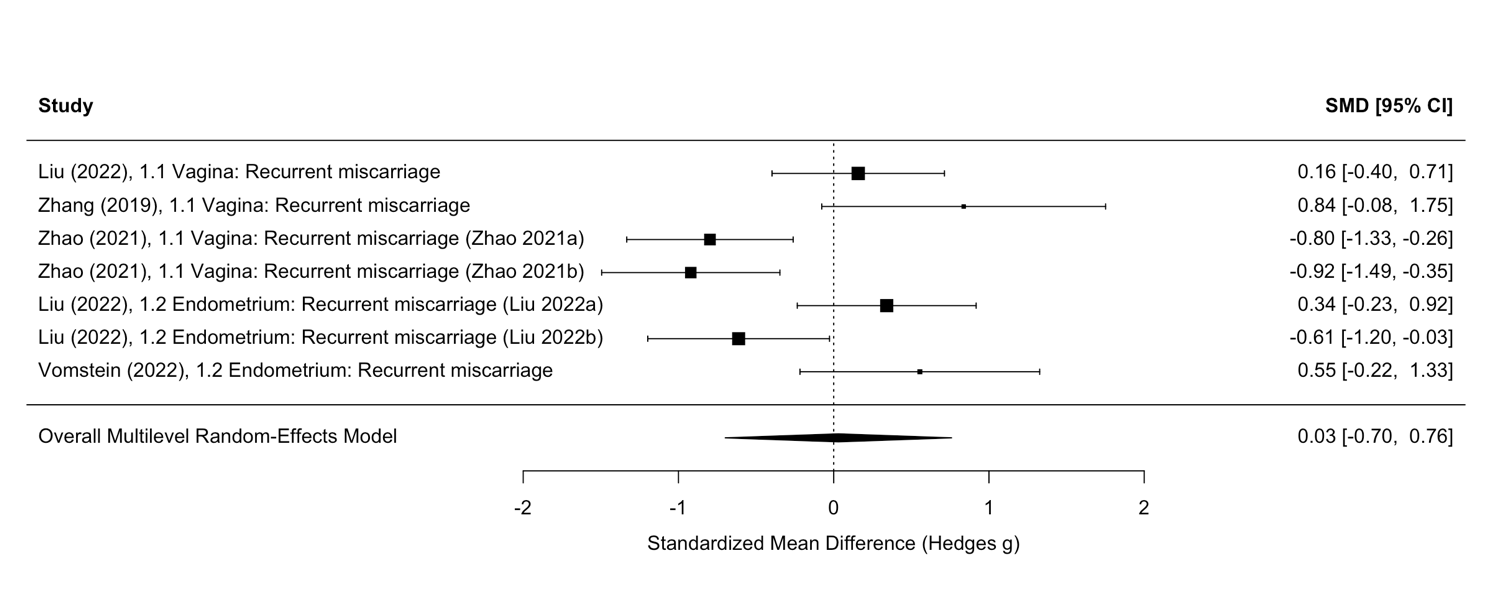
***.

| **8c: Summary statistics for forest plots** | | | | | | | |
| --- | --- | --- | --- | --- | --- | --- | --- |
| **Model** | **k** | **SMD Estimate** | **95% CI** | **p-value** | **Q(df), p** | **σ²₁ (Between-study)** | **σ²₂ (Within-study)** |
| **Shannon diversity recurrent** | 12 | –0.15 | –0.57 to 0.26 | 0.47 | Q(11) = 39.57, p < .001 | 0.041 | 0.287 |
| **Chao1 diversity recurrent** | 7 | 0.03 | –0.70 to 0.76 | 0.94 | Q(6) = 26.34, p = .0002 | 0.403 | 0.082 |

### **Supplementary figures 9:** Funnel plots for meta-analyses (9a: Relative abundance of *Lactobacillus*, 9b: Relative abundance of *Lactobacillus* reporting ≥70% STORMS checklist, 9c: Shannon index in sporadic miscarriage, 9d: Chao in sporadic miscarriage, 9e: Observed species in sporadic miscarriage, 9f: Shannon index in recurrent miscarriage, 9g: Chao in recurrent miscarriage)

**Figure 9a: Funnel plot for relative abundance of *Lactobacillus***

*
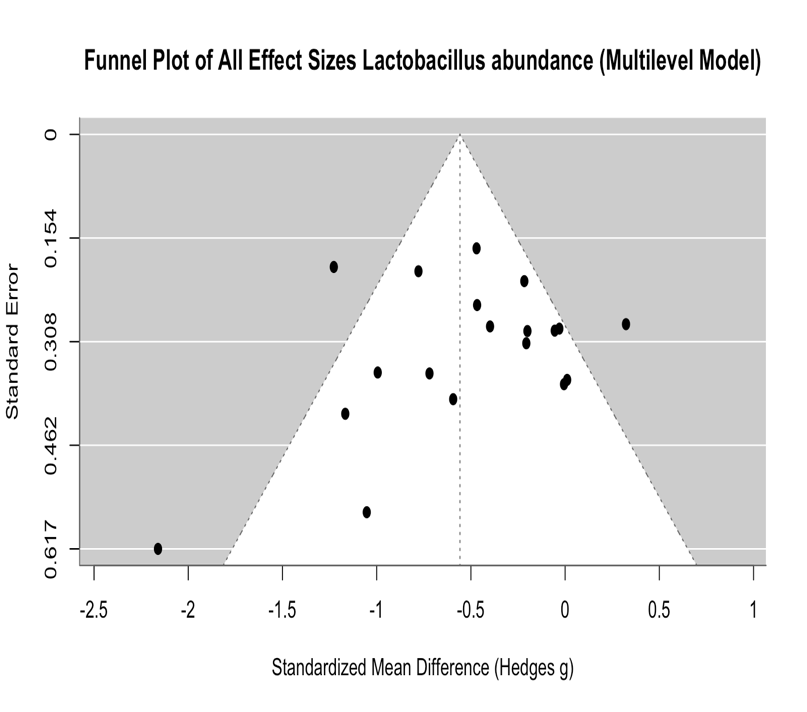
*

**Figure 9b: Funnel plot for relative abundance of *Lactobacillus* reporting ≥70% STORMS checklist**

**
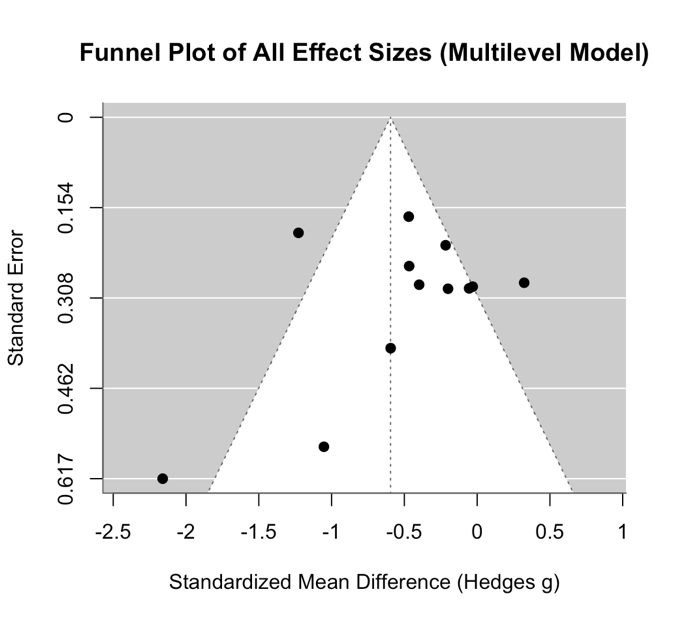
**

**Figure 9c: Funnel plot for Shannon index in sporadic miscarriage**

**
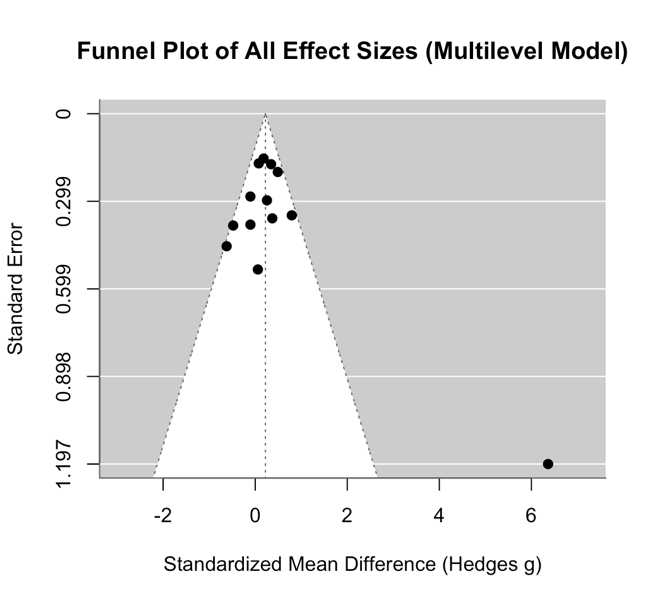
**

**Figure 9d: Funnel plot for Chao in sporadic miscarriage**

**
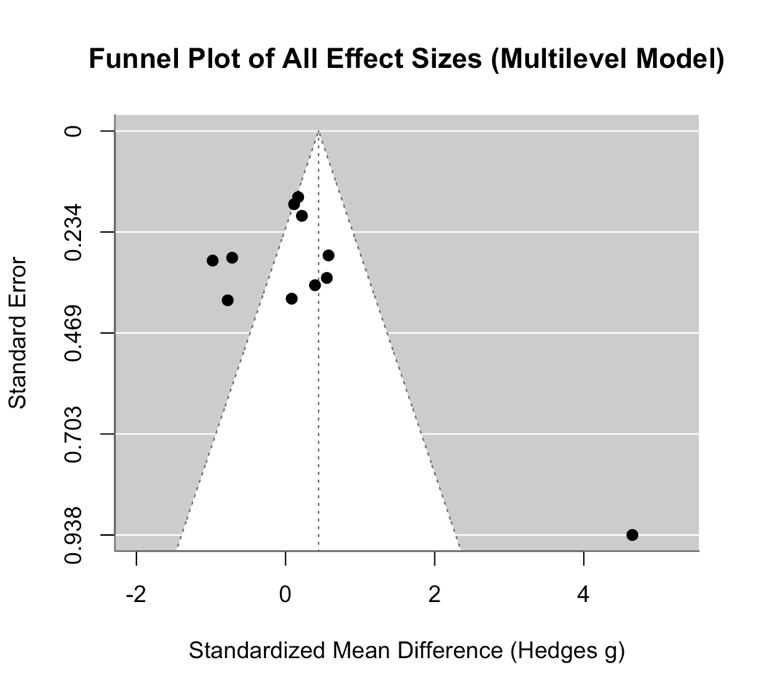
**

**Figure 9e: Funnel plot for Observed species in sporadic miscarriage**

**
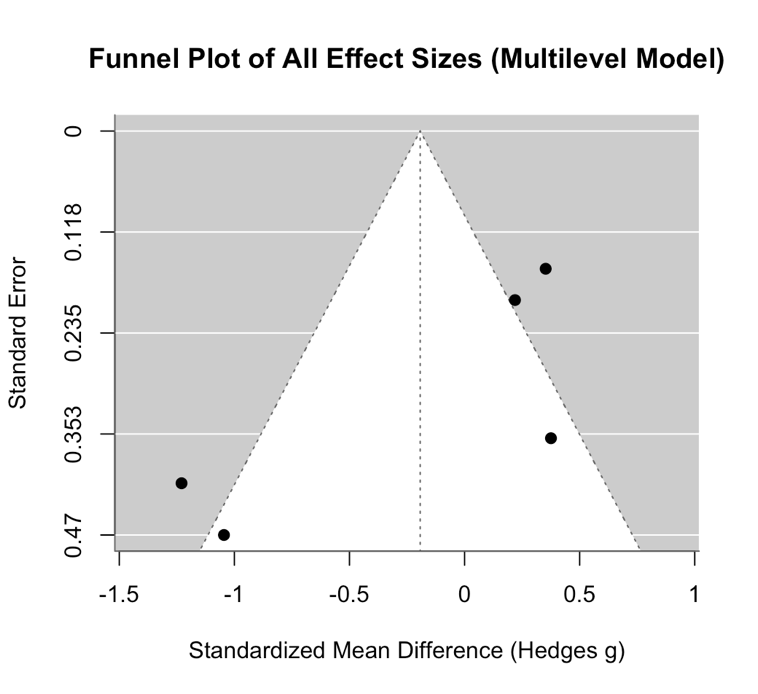
**

| ***Egger’s test*** | |
| --- | --- |
| *Intercept* | *-3.9262* |
| *Standard error* | *1.6994* |
| *Significance level* | *p = 0.1040* |

**Figure 9f: Funnel plot for Shannon index in recurrent miscarriage**

**
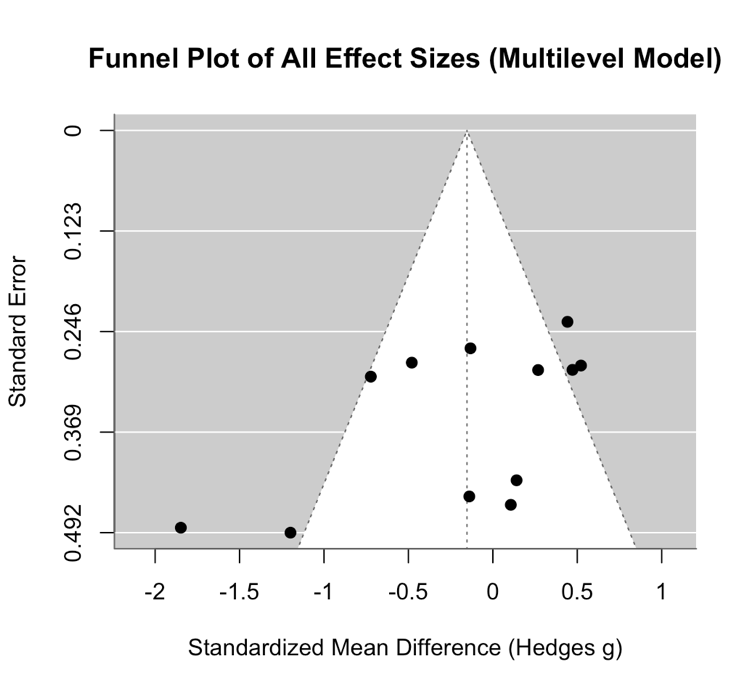
**

**Figure 8g: Funnel plot for Chao in recurrent miscarriage**

**
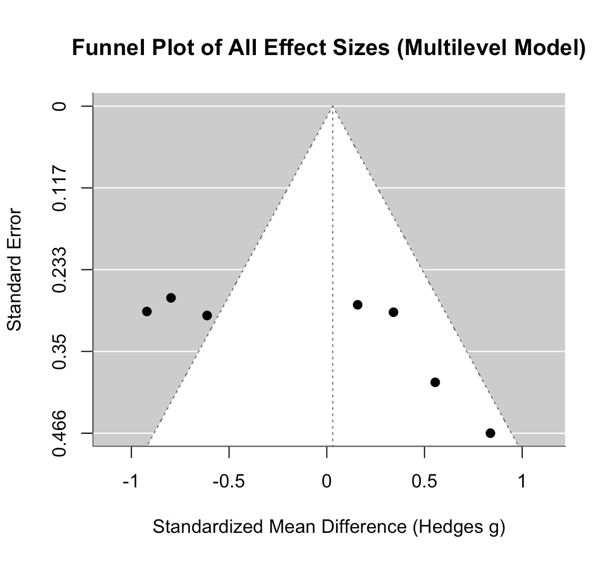
**

| **Supplementary table 10.** Summary of beta diversity measures | | | | | |
| --- | --- | --- | --- | --- | --- |
| **Author (Year)** | **Body Site** | **Sporadic miscarriage (S) or Recurrent miscarriage (R)** | **Metric** | **Analysis** | **Finding** |
| Bai (2024) | Endometrium | R | Bray-curtis | PCA | No results from controls for comparison |
| Chen (2022) | Vagina | S | Binary Jaccard | PCoA | Sig. difference |
|  |  |  | Unweighted UniFrac | PCoA | Sig. difference |
| Fan (2020) | Vagina | R | Unweighted UniFrac | PCoA | Sig. difference |
| Fernandez (2021) | Vagina | R | Bray-Curtis | PCoA, PERMANOVA | Sig. difference |
|  |  |  | Binary Jaccard | PCoA, PERMANOVA | Sig. difference |
| Goncharov (2021) | Vagina | S | Unweighted UniFrac | PCoA | Sig. difference |
| Gryaznova (2023) | Cervix | S | Bray-Curtis | PCoA, NMDS | No sig. difference |
|  | Vagina | S | Bray-Curtis | PCoA, NMDS | No sig. difference |
| Gryaznova (2024) | Cervix | S | Bray-Curtis | NR | No sig. difference |
| Guang (2022) | Cervix | S | Weighted UniFrac | PCoA | No sig. difference |
|  | Vagina | S | Weighted UniFrac | PCoA | No sig. difference |
| Han (2019) | Endometrium (Miscarriage vs TOP) | S | Unweighted UniFrac | PCoA | Sig. difference |
|  | Endometrium (Miscarriage vs not pregnant) | S | Unweighted UniFrac | PCoA | No sig. difference |
| Jiao (2022) | Vagina | R | Bray-Curtis | PCoA PLS-DA | No sig. difference No sig. difference |
| Liu (2021) | Vagina (Missed miscarriage vs normal pregnancy) | S | Unweighted UniFrac | PCoA | Sig. difference |
|  | Vagina (Empty sac miscarriage vs normal pregnancy) | S | Unweighted UniFrac | PCoA | No sig. difference |
| Liu (2022) | Cervix | R | Bray-Curtis | PCoA Permanova | No sig. difference  Sig. difference (Cases up) |
|  | Endometrial tissue | R | Bray-Curtis | PCoA PERMANOVA | No sig. difference No sig. difference |
|  | Uterine lavage | R | Bray-Curtis | PCoA PERMANOVA | No sig. difference Sig. difference (Cases up) |
|  | Vagina | R | Bray-Curtis | PCoA PERMANOVA | No sig. difference Sig. difference (Cases up) |
| Liu (2025) | Endometrium | S | Weighted UniFrac | ANOSIM, PCA | Sig. difference |
| Mauscci (2023) | Endometrium (HLA-DQ2/DQ8 pos vs HC) | R | Weighted UniFrac | PCoA | Sig. difference |
|  | Endometrium (HLA-DQ2/DQ8 neg vs HC) | R | Weighted UniFrac | PCoA | No sig. difference |
|  | Vagina (All groups) | R | Weighted UniFrac | PCoA | No sig. difference |
| McClelland (2024) | Vagina (Peri-conception sampling of first trimester miscarriage vs peri-conception sampling of ongoing pregnancy)  Vagina (First trimester sampling of first trimester miscarriage vs first trimester sampling of ongoing pregnancy) | S  S | Relative abundance measures  Relative abundance measures | PCA  PCA | No sig. difference  No sig. difference |
| Peuranpaa (2022) | Vagina | R | - | PERMANOVA | No sig. difference |
|  | Endometrium | R | - | PERMANOVA | Sig. difference |
| Severgnini (2022) | Vagina | S | Weighted UniFrac | PCoA | No sig. difference |
|  | Vagina | S | Unweighted UniFrac | PCoA | No sig. difference |
| Shahid (2022) | Vagina | S | Bray-Curtis | nMDS PERMDISP | No sig. difference |
| Shu (2022) | Endometrium | S | Weighted  Unweighted | nMDS | No sig. difference |
| Skafte-Holm (2025) | Vagina | S | - | PCoA  PERMANOVA | No sig. difference |
| Sun (2022) | Vagina | S | Unweighted UniFrac Weighted UniFrac Bray-Curtis | PCoA | Sig. difference |
| Tan (2024) | Vagina | R | Binary Jaccard  Bray-Curtis  Euclidean distance | PCoA  PERMANOVA  NMDS | Sig. difference |
| Vomstein (2022) | Endometrium | R | Bray-Curtis Weighted UniFrac Unweighted UniFrac | PCoA MDS PERMANOVA | Sig. difference |
| Wang (2023) | Vagina | S | Bray-Curtis Unweighted UniFrac | PCoA | Sig. difference |
|  | Endometrium | S | Bray-Curtis Unweighted UniFrac | PCoA | Sig. difference |
| Wang (2025) | Vagina | S/R | Bray-Curtis (Species) | PCoA  PERMANOVA | Sig. difference |
|  |  |  | Bray-Curtis (Genus) | PCoA  PERMANOVA | No sig. difference |
| Wei (2024) | Endometrium | S | Bray-Curtis | PCoA | No sig. difference |
| Xu (2020) | Vagina | S | - | NMDS PCoA PCA | No sig. difference |
| Zhang (2019) | Vagina | R | Bray-Curtis Weighted UniFrac | PCoA | No sig. difference |
| Zhao (2021) | Vagina | R | Unweighted UniFrac | PCoA MANOVA | Sig. difference |
|  | Vagina | R | Weighted UniFrac | PCoA MANOVA | No sig. difference |

### **Supplementary table 11:** Excluded studies after full text review

| **Title** | **Author Year** | **Journal** | **Vol** | **Page** | **Notes** |
| --- | --- | --- | --- | --- | --- |
| The influence of vaginal microbiota on frozen blastocyst implantation after transfer: a prospective study through nextgeneration 16S rrna sequencing | Asakura 2021 | Human Reproduction | 36 | i56-i57 | Exclusion reason: Wrong outcomes |
| Characterizing reproductive tract microbiome through shotgun metagenomic sequencing at time of ivf, a pilot study | Bardos 2021 | Fertility and sterility | 116 | e112 | Exclusion reason: Wrong comparator; |
| Establishment of vaginal microbiota composition in early pregnancy and its association with subsequent preterm prelabor rupture of the fetal membranes | Brown 2019 | Translational Research | 207 | 30-43 | Exclusion reason: Wrong outcomes; |
| The uterine microbiome in recurrent pregnancy loss | Churchill 2018 | Fertility and sterility | 109 | e12 | Exclusion reason: Wrong study design; |
| Association of the vaginal microbiome with prophylactic antibiotic exposure and clinical outcomes in women undergoing in vitro fertilization: a randomized controlled pilot study | Eskew 2019 | Fertility and sterility | 112 | e183 | Exclusion reason: Wrong study design; |
| The relationship between systemic oestradiol and vaginal microbiota composition in miscarriage and normal pregnancy | Fourie 2021 | Human Reproduction | 36 | i311-i312 | Exclusion reason: Wrong outcomes |
| An Altered Endometrial Microbiome Activates Immune System and Defense Response in the Endometrium of Women with Reproductive Failure after IVF | Gonzalez-Monfort 2023 | Reproductive Sciences | 30 | 86A | Exclusion reason: Wrong outcomes; |
| The endometrial microbiome of clinical miscarriage, ectopic pregnancy and during early pregnancy in a successful livebirth | Grau 2019 | Fertility and sterility | 112 | e315-e316 | Exclusion reason: Case report or case series; |
| Euploid miscarriage is associated with lactobacillus spp. Deplete vaginal microbial composition and local inflammation | Grewal 2020 | Reproductive Sciences | 27 | 65A | Exclusion reason: Duplicate; |
| Does dysbiotic endometrium affect blastocyst implantation in IVF patients? | Hashimoto 2019 | Journal of assisted reproduction and genetics | 36 | 2471-2479 | Exclusion reason: Wrong comparator; |
| Efficacy of endometrial microbiome metagenomic analysis and analysis of infectious chronic endometritis on in vitro fertilization outcome in women with recurrent implantation failure | Iwami 2020 | Human Reproduction | 35 | i31 | Exclusion reason: Wrong study design; |
| Therapeutic intervention based on gene sequencing analysis of microbial 16S ribosomal RNA of the intrauterine microbiome improves pregnancy outcomes in IVF patients: a prospective cohort study | Iwami 2023 | Journal of assisted reproduction and genetics | 40 | 125-135 | Exclusion reason: Wrong study design; |
| Efficacy of endometrial microbiome metagenomic analysis with recurrent implantation failure and recurrent pregnancy loss: multicenter study in Japan | Iwami 2022 | Human Reproduction | 37 | i95 | Exclusion reason: Wrong study design; |
| Characterization of vaginal microbiota related with high-risk pregnancy in Korea | Kim 2018 | American Journal of Reproductive Immunology | 80 | 88-89 | Exclusion reason: Wrong study design; |
| Higher incidence of colonization with gardnerella vaginalis and gram-negative anaerobes in patients with recurrent miscarriage and elevated peripheral natural killer cells | Kuon 2017 | Human Reproduction | 32 | i344 | Exclusion reason: Does not use Next Generation Sequencing (NGS); |
| Analysis of endometrial microbiota by 16S ribosomal RNA gene sequencing among infertile patients: a single-center pilot study | Kyono 2018 | Reproductive Medicine and Biology | 17 | 297-306 | Exclusion reason: Wrong study design; |
| Endometrial microbiota and pregnancy outcome of IVF patients in Japan: an analysis using species level resolution 16S rrna gene amplicon sequencing | Kyono 2019 | Human Reproduction | 34 | i120 | Exclusion reason: Wrong patient population; |
| Systematic Comparison of Bacterial Colonization of Endometrial Tissue and Fluid Samples in Recurrent Miscarriage Patients: Implications for Future Endometrial Microbiome Studies | Liu 2018 | Clinical chemistry | 64 | 1743-1752 | Exclusion reason: Wrong study design; |
| Vaginal microbiocenosis in various clinical forms of miscarriage | Lobanova 2019 | Obstetrics, Gynecology and Reproduction | 13 | 13-19 | Exclusion reason: Does not use Next Generation Sequencing (NGS); |
| Composition of the endometrial microbiome is associated to reproductive outcomes in IVF patients | Moreno 2020 | Human Reproduction | 35 | i31-i32 | Exclusion reason: Wrong comparator; |
| Endometrial microbiota composition is associated with reproductive outcome in infertile patients | Moreno 2021 | Human Reproduction | 36 | i54-i55 | Exclusion reason: Wrong study design; |
| The investigation of pathogenic bacteria in female genital flora of patients with recurrent pregnancy lass by I6S rrna gene sequencing | Mori 2019 | Journal of Obstetrics and Gynaecology Research | 45 | 1713 | Exclusion reason: Wrong study design; |
| Torquetenovirus in pregnancy: Correlation with vaginal microbiome, metabolome and pro-inflammatory cytokines | Morselli 2022 | Frontiers in microbiology | 13 | 998849 | Exclusion reason: Wrong outcomes; |
| Influence of Pregnancy History on the Vaginal Microbiome of Pregnant Women in their First Trimester | Nasioudis 2017 | Scientific reports | 7 | 10201 | Exclusion reason: Wrong outcomes; |
| High Prevalence of Leptotrichia amnionii, Atopobium vaginae, Sneathia sanguinegens, and Factor 1 Microbes and Association of Spontaneous Abortion among Korean Women | Seo 2017 | BioMed research international | 2017 | 5435089 | Exclusion reason: Wrong patient population; |
| Anamnestic and microbiological predictors of miscarriage | Sinyakova 2019 | Journal of Obstetrics and Women's Diseases | 68 | 59-70 | Exclusion reason: Does not use Next Generation Sequencing (NGS); |
| Derangements of vaginal and cervical canal microbiota determined with real-time PCR in women with recurrent miscarriages | SoyerCaliskan 2022 | Journal of obstetrics and gynaecology : the journal of the Institute of Obstetrics and Gynaecology | 42 | 2105-2114 | Exclusion reason: Does not use Next Generation Sequencing (NGS); |
| Vaginal microbiota profile at the time of embryo transfer does not affect live birth rate in IVF cycles with donated oocytes | Vergaro 2019 | Reproductive biomedicine online | 38 | 883-891 | Exclusion reason: Does not use Next Generation Sequencing (NGS); |
| A retrospective pilot study to determine whether the reproductive tract microbiota differs between women with a history of infertility and fertile women | Wee 2018 | The Australian & New Zealand journal of obstetrics & gynaecology | 58 | 341-348 | Exclusion reason: Wrong outcomes; |
| Non-Lactobacillus dominance of the vagina is associated with reduced live birth rate following IVF/ICSI: a propensity score-matched cohort study | Zeng 2022 | Archives of gynecology and obstetrics | 305 | 519-528 | Exclusion reason: Does not use Next Generation Sequencing (NGS); |
| The endometrial microbiota profile influenced pregnancy outcomes in patients with repeated implantation failure: a retrospective study | Zou 2023 | Journal of reproductive immunology | 155 | 103782 | Exclusion reason: Wrong intervention; |
| Microbial Diversity and Pathogenic Properties of Microbiota Associated with Aerobic Vaginitis in Women with Recurrent Pregnancy Loss | Ncib 2022 | Diagnostics (Basel) | 12 |  | Exclusion reason: Does not use Next Generation Sequencing (NGS); |
| Endometrial microbiome: sampling, assessment, and possible impact on embryo implantation | Reschini 2022 | Sci Rep | 12 | 8467 | Exclusion reason: Wrong outcomes; |
| Bacterial vaginosis in a subfertile population undergoing fertility treatments: a prospective cohort study | VanDenTweel 2023 | Human Reproduction | 38 | i419 | Exclusion reason: Wrong outcomes; |
| The vaginal and faecal microbiome in women with recurrent pregnancy loss (RPL) before pregnancy according to the reproductive outcome after referral | Krog 2023 | Human Reproduction | 38 | i160 | Exclusion reason: Wrong outcomes; |
| The influence of the vaginal microbiome on clinical outcomes in patients undergoing a frozen embryo transfer: a prospective pilot study | Povey 2023 | Human Reproduction | 38 | i159 | Exclusion reason: Wrong outcomes; |
| Characterization of vaginal microbiota during IVF fresh embryo transfer (IVF-ET) and in early pregnancy | Vainamo 2023 | Human Reproduction | 38 | i157-i158 | Exclusion reason: Wrong outcomes; |
| NGS-based metagenome analysis of endometrial microbiome in women with implantation failure after in vitro fertilization: results of a prospective cohort study | Hadjidekova 2023 | Human Reproduction | 38 | i169-i170 | Exclusion reason: Wrong outcomes; |
| Higher prevalence of colonization with <i>Gardnerella vaginalis and gram-negative anaerobes</i> in patients with recurrent miscarriage and elevated peripheral natural killer cells | Kuon 2017 | JOURNAL OF REPRODUCTIVE IMMUNOLOGY | 120 | 15-19 | Exclusion reason: Does not use Next Generation Sequencing (NGS); |
| Association of spontaneous abortion and<i>Ureaplasma parvum</i>detected in placental tissue | Oliveira 2020 | EPIDEMIOLOGY AND INFECTION | 148 |  | Exclusion reason: Does not use Next Generation Sequencing (NGS); |
| Exploring the Role of Lower Genital Tract Microbiota and Cervical-Endometrial Immune Metabolome in Unknown Genesis of Recurrent Pregnancy Loss. | Mikhalev 2025 | International journal of molecular sciences | 26 |  | Exclusion reason: Does not use Next Generation Sequencing (NGS); |
| Cervicovaginal microbiome and natural history of Chlamydia trachomatis in adolescents and young women. | Usyk 2025 | Cell | 188 | 1051-1061.e12 | Exclusion reason: Wrong outcomes; |
| Differential characteristics of vaginal versus endometrial microbiota in IVF patients. | Polifke 2024 | Scientific reports | 14 | 30508 | Exclusion reason: Wrong comparator; |
| A Cross-Sectional Comparative Study of Vaginal Microbiota and Spontaneous Abortion at a Tertiary Care Hospital in North Karnataka, India. | Belgundkar 2024 | Nursing for women's health | 28 | 375-380 | Exclusion reason: Does not use Next Generation Sequencing (NGS); |
| Assessing vaginal microbiome through Vaginal Microecology Evaluation System as a predictor for in vitro fertilization outcomes: a retrospective study. | Tian 2024 | Frontiers in endocrinology | 15 | 1380187 | Exclusion reason: Does not use Next Generation Sequencing (NGS); |
| Characteristics of vaginal microbiota in pregnant women with cervical insufficiency and its association with adverse pregnancy outcomes | Zhang 2025 | Pakistan Journal of Medical Sciences | 41 | 1381EP - 1387 | Exclusion reason: Wrong comparator; |
| Comparison between real-time PCR method (Flora selecttm) and Nugent's score for the diagnosis of bacterial vaginosis during pregnancy | Yamada 2025 | Journal of Reproductive Immunology | 169 | 104493 | Exclusion reason: Does not use Next Generation Sequencing (NGS); |
| The influence and association of blood on transfer catheter and vaginal microbiota on pregnancy outcomes in frozen-embryo transfer cycles | Fu 2025 | Journal of Reproductive Immunology | 169 | 104530 | Exclusion reason: Wrong comparator; |
| Higher Incidence of Colonization with Gardnerella Vaginalis in Patients with Recurrent Miscarriage and Elevated Peripheral Natural Killer Cells | Kuon 2017 | Journal of Perinatal Medicine | 45 | 21 | Exclusion reason: Does not use Next Generation Sequencing (NGS); |
| "Shortening time to pregnancy in infertile women by personalizing treatment of microbial imbalance through Emma & Alice: A multicenter prospective study" | Iwami 2025 | Reproductive Medicine and Biology | 24 | e12634 | Exclusion reason: Wrong outcomes; |
| Prognostic significance of the microbial factor in patients with miscarriage in early pregnancy registration | Beliaeva 2024 | Journal of Obstetrics and Women's Diseases | 73 | 32EP - 43 | Exclusion reason: Does not use Next Generation Sequencing (NGS); |
| Microbe-binding Antibodies in the Female Genital Tract: Associations with the Vaginal Microbiome and Genital Immunology | Liu 2024 | Journal of Immunology | 213 | 1516EP - 1527 | Exclusion reason: Wrong study design; |
| Utility of culture and molecular methods using allplextm Bacterial Vaginosis Plus Assay (Seegene) as a tool for endometriosis, infertility and recurrent pregnancy loss diagnosis | Maldonado-Barrueco 2024 | Diagnostic Microbiology and Infectious Disease | 110 | 116437 | Exclusion reason: Does not use Next Generation Sequencing (NGS); |
| An intervention according to the results of a new endometrial microbiome testing improves art outcomes in recurrent implantation failure patients | Takeuchi 2024 | Fertility and Sterility | 122 | e219 | Exclusion reason: Wrong study design; |
| An exploratory study of endometrial dysbiosis in recurrent early pregnancy loss and associated reproductive outcomes | Patel 2024 | Fertility and Sterility | 122 | e183 | Exclusion reason: Wrong outcomes; |
| Characterization of Vaginal microbiota and reproductive outcomes associated with assisted reproductive technologies through next-generation sequencing, from Indian population | Vajpeyee 2024 | Human Reproduction | 39 | i161 | Exclusion reason: Wrong outcomes; |
| Effect of vaginal microbiota disorder on pregnancy outcomes in frozen-thawed embryo transfer patients: a retrospective cohort study | Liu 2024 | Chinese Journal of Reproduction and Contraception | 44 | 456EP - 462 | Exclusion reason: Wrong study design; |
| Spatial Localization of Eubacterial 16S rrna in Early Pregnancy Placenta and Decidua | Thoeni 2024 | Pediatric and Developmental Pathology | 27 | 132EP - 138 | Exclusion reason: Wrong study design; |
| Isolation and Characterization of Streptococcus agalactiae and its Capsular Antigen, Along with Mycoplasma hominis and Listeria monocytogenes, as Abundant Infections in Women with Abortion in Iran | Bayat 2023 | Jundishapur Journal of Microbiology | 16 | e141748 | Exclusion reason: Does not use Next Generation Sequencing (NGS); |
| Longitudinal analysis of vaginal microbiota during IVF fresh embryo transfer and in early pregnancy | Vainamo 2023 | Microbiology Spectrum | 11 |  | Exclusion reason: Wrong outcomes; |
| Prevalence of Pathogenic Microbes within the Endometrium in Normal Weight vs. Obese Women with Infertility | King 2024 | REPRODUCTIVE MEDICINE | 5 | 90-96 | Exclusion reason: Wrong patient population; |
| Molecular Diagnosis of Vaginal Microbiota Associated with Spontaneous Abortion in Women | Al-Hajjar 2024 | Iraqi Journal of Science | 65 | 5474-5787 | Exclusion reason: Does not use Next Generation Sequencing (NGS); |
| Vaginal Microflora Characteristics in Women at Risk of Miscarriage and Those with a Normal Course of Pregnancy, Serving as a Potential Determinant of Pregnancy Outcomes | Koshulko 2024 | Reproductive Health Eastern Europe | 14 | 467-477 | Exclusion reason: Does not use Next Generation Sequencing (NGS); |
| Changes in the Vaginal Microbiome During Pregnancy and the Postpartum Period in South African Women: a Longitudinal Study | Li 2024 | Reproductive Sciences | 31 | 275-287 | Exclusion reason: Wrong outcomes; |
| The relationship between vaginal flora changes and spontaneous abortion in early pregnancy at advanced age | He 2023 | Chinese Journal of Microecology | 35 | 322-326and330 | Exclusion reason: Does not use Next Generation Sequencing (NGS); |

### **Supplementary table 12:** PRISMA checklist

| **Section and Topic** | **Item #** | **Checklist item** | **Location where item is reported** |
| --- | --- | --- | --- |
| **TITLE** | | |  |
| Title | 1 | Identify the report as a systematic review. | Pg 1 |
| **ABSTRACT** | | |  |
| Abstract | 2 | See the PRISMA 2020 for Abstracts checklist. | Pg 2 – note reported according to journal requirements not PRISMA. |
| **INTRODUCTION** | | |  |
| Rationale | 3 | Describe the rationale for the review in the context of existing knowledge. | Pg 3, para 1-3 |
| Objectives | 4 | Provide an explicit statement of the objective(s) or question(s) the review addresses. | Pg 3-4, para 3 |
| **METHODS** | | |  |
| Eligibility criteria | 5 | Specify the inclusion and exclusion criteria for the review and how studies were grouped for the syntheses. | Pg 12, section ‘Selection criteria’. |
| Information sources | 6 | Specify all databases, registers, websites, organisations, reference lists and other sources searched or consulted to identify studies. Specify the date when each source was last searched or consulted. | Pg 12, section ‘Search details’ |
| Search strategy | 7 | Present the full search strategies for all databases, registers and websites, including any filters and limits used. | Supplementary table 1 |
| Selection process | 8 | Specify the methods used to decide whether a study met the inclusion criteria of the review, including how many reviewers screened each record and each report retrieved, whether they worked independently, and if applicable, details of automation tools used in the process. | Pg 12, section ‘Selection criteria’. |
| Data collection process | 9 | Specify the methods used to collect data from reports, including how many reviewers collected data from each report, whether they worked independently, any processes for obtaining or confirming data from study investigators, and if applicable, details of automation tools used in the process. | Pg 12, section ‘Data extraction’. |
| Data items | 10a | List and define all outcomes for which data were sought. Specify whether all results that were compatible with each outcome domain in each study were sought (e.g. for all measures, time points, analyses), and if not, the methods used to decide which results to collect. | Pg 12, section ‘Data extraction’, |
|  | 10b | List and define all other variables for which data were sought (e.g. participant and intervention characteristics, funding sources). Describe any assumptions made about any missing or unclear information. | Pg 12, section ‘Data extraction’. |
| Study risk of bias assessment | 11 | Specify the methods used to assess risk of bias in the included studies, including details of the tool(s) used, how many reviewers assessed each study and whether they worked independently, and if applicable, details of automation tools used in the process. | Pg 13, section ‘Quality assessment’. |
| Effect measures | 12 | Specify for each outcome the effect measure(s) (e.g. risk ratio, mean difference) used in the synthesis or presentation of results. | Pg 13, section ‘Data synthesis’ |
| Synthesis methods | 13a | Describe the processes used to decide which studies were eligible for each synthesis (e.g. tabulating the study intervention characteristics and comparing against the planned groups for each synthesis (item #5)). | Pg 12, section ‘Data extraction’, paragraph 2 and pg 13, section ‘Data synthesis’. |
|  | 13b | Describe any methods required to prepare the data for presentation or synthesis, such as handling of missing summary statistics, or data conversions. | Pg 12, section ‘Data extraction’ paragraph 2. |
|  | 13c | Describe any methods used to tabulate or visually display results of individual studies and syntheses. | Pg 13, section ‘Data synthesis’. |
|  | 13d | Describe any methods used to synthesize results and provide a rationale for the choice(s). If meta-analysis was performed, describe the model(s), method(s) to identify the presence and extent of statistical heterogeneity, and software package(s) used. | Pg 13, section ‘Data synthesis’. |
|  | 13e | Describe any methods used to explore possible causes of heterogeneity among study results (e.g. subgroup analysis, meta-regression). | Pg 13, section ‘Data synthesis’ |
|  | 13f | Describe any sensitivity analyses conducted to assess robustness of the synthesized results. | Pg 13, section ‘Data synthesis’. |
| Reporting bias assessment | 14 | Describe any methods used to assess risk of bias due to missing results in a synthesis (arising from reporting biases). | Not performed but limitations discussed (pg 10, discussion’. |
| Certainty assessment | 15 | Describe any methods used to assess certainty (or confidence) in the body of evidence for an outcome. | Not performed |
| **RESULTS** | | |  |
| Study selection | 16a | Describe the results of the search and selection process, from the number of records identified in the search to the number of studies included in the review, ideally using a flow diagram. | Figure 1 |
|  | 16b | Cite studies that might appear to meet the inclusion criteria, but which were excluded, and explain why they were excluded. | Supplementary table 11 |
| Study characteristics | 17 | Cite each included study and present its characteristics. | Table 1 |
| Risk of bias in studies | 18 | Present assessments of risk of bias for each included study. | Supplementary table 2 and 3 |
| Results of individual studies | 19 | For all outcomes, present, for each study: (a) summary statistics for each group (where appropriate) and (b) an effect estimate and its precision (e.g. confidence/credible interval), ideally using structured tables or plots. | Table 1, figure 5, supplementary figures 6, 7, 8 |
| Results of syntheses | 20a | For each synthesis, briefly summarise the characteristics and risk of bias among contributing studies. | This can be cross-referenced to table 1. |
|  | 20b | Present results of all statistical syntheses conducted. If meta-analysis was done, present for each the summary estimate and its precision (e.g. confidence/credible interval) and measures of statistical heterogeneity. If comparing groups, describe the direction of the effect. | Figure 5, supplementary figures 6, 7, 8. |
|  | 20c | Present results of all investigations of possible causes of heterogeneity among study results. | Not applicable |
|  | 20d | Present results of all sensitivity analyses conducted to assess the robustness of the synthesized results. | Supplementary figures 6, pg 6, section ‘Assessment of alterations in FRT microbiome’ para 3. |
| Reporting biases | 21 | Present assessments of risk of bias due to missing results (arising from reporting biases) for each synthesis assessed. | Not applicable |
| Certainty of evidence | 22 | Present assessments of certainty (or confidence) in the body of evidence for each outcome assessed. | Not applicable |
| **DISCUSSION** | | |  |
| Discussion | 23a | Provide a general interpretation of the results in the context of other evidence. | Pg 9-10, discussion para 2, 3, 4 |
|  | 23b | Discuss any limitations of the evidence included in the review. | Pg 11 para 2 |
|  | 23c | Discuss any limitations of the review processes used. | Pg 11, para 2 |
|  | 23d | Discuss implications of the results for practice, policy, and future research. | Pg 9, discussion para 2 and pg 11, para 3 |
| **OTHER INFORMATION** | | |  |
| Registration and protocol | 24a | Provide registration information for the review, including register name and registration number, or state that the review was not registered. | Pg 11, section ‘Methods’. |
|  | 24b | Indicate where the review protocol can be accessed, or state that a protocol was not prepared. | PROSPERO registration stated so can be accessed through search of this. |
|  | 24c | Describe and explain any amendments to information provided at registration or in the protocol. | Not applicable |
| Support | 25 | Describe sources of financial or non-financial support for the review, and the role of the funders or sponsors in the review. | Pg 14, section ‘Acknowledgements’. |
| Competing interests | 26 | Declare any competing interests of review authors. | Pg 14, section ‘Competing interests’. |
| Availability of data, code and other materials | 27 | Report which of the following are publicly available and where they can be found: template data collection forms; data extracted from included studies; data used for all analyses; analytic code; any other materials used in the review. | Pg 13, section ‘Data availability’ and ‘Code availability’. |

*From:*  Page MJ, McKenzie JE, Bossuyt PM, Boutron I, Hoffmann TC, Mulrow CD, et al. The PRISMA 2020 statement: an updated guideline for reporting systematic reviews. BMJ 2021;372:n71. doi: 10.1136/bmj.n71. This work is licensed under CC BY 4.0. To view a copy of this license, visit <https://creativecommons.org/licenses/by/4.0/>

### **Supplementary tables 13-18:** Summary of individual microbial changes **(see separate Excel document)**

#### Supplementary table 13: Summary of findings for each taxon reported at phylum level for VAGINAL samples

#### Supplementary table 14: Summary of findings for each taxon reported at phylum level for CERVICAL samples

#### Supplementary table 15: Summary of findings for each taxon reported at phylum level for ENDOMETRIAL samples

#### Supplementary table 16: Summary of findings for each taxon reported at genus level for VAGINAL samples

#### Supplementary table 17: Summary of findings for each taxon reported at genus level for CERVICAL samples

#### Supplementary table 18: Summary of findings for each taxon reported at genus level for ENDOMETRIAL samples

***Key***

| ↑ | Statistically significant increase in cases compared to controls |
| --- | --- |
| *↓* | Statistically significant decrease in cases compared to controls |
|  | No difference between cases and controls |
|  | Individual phyla/genus was present in samples but no statistical comparison was presented in the paper |

1. Al-Memar M, Bobdiwala S, Fourie H, Mannino R, Lee YS, Smith A, et al. The association between vaginal bacterial composition and miscarriage: a nested case-control study. Bjog-an International Journal of Obstetrics and Gynaecology. 2020;127(2):264-74.

2. Barinova VV, Kuznetsova NB, Bushtyreva IO, Dudurich VV, Shatalov AE. Uterine microbiome and immunohistochemical markers of chronic endometritis in recurrent pregnancy loss. Obstetrics and Gynecology. 2022(4):84-94.

3. Bui BN, van Hoogenhuijze N, Viveen M, Mol F, Teklenburg G, de Bruin J-P, et al. The endometrial microbiota of women with or without a live birth within 12 months after a first failed IVF/ICSI cycle. Scientific Reports. 2023;13(1):3444.

4. Chang DH, Shin J, Rhee MS, Park KR, Cho BK, Lee SK, et al. Vaginal Microbiota Profiles of Native Korean Women and Associations with High-Risk Pregnancy. Journal of Microbiology and Biotechnology. 2020;30(2):248-58.

5. Chen S, Xue XM, Zhang YX, Zhang HM, Huang XG, Chen XF, et al. Vaginal Atopobium is Associated with Spontaneous Abortion in the First Trimester: a Prospective Cohort Study in China. Microbiology Spectrum. 2022;10(2).

6. Fan T, Zhong XM, Wei XC, Miao ZL, Luo SY, Cheng H, et al. The alteration and potential relationship of vaginal microbiota and chemokines for unexplained recurrent spontaneous abortion. Medicine (Baltimore). 2020;99(51):e23558.

7. Fernández L, Castro I, Arroyo R, Alba C, Beltrán D, Rodríguez JM. Application of Ligilactobacillus salivarius CECT5713 to Achieve Term Pregnancies in Women with Repetitive Abortion or Infertility of Unknown Origin by Microbiological and Immunological Modulation of the Vaginal Ecosystem. Nutrients. 2021;13(1).

8. A. E. Goncharov SVR, B. I. Aslanov, E. A. Lebedeva et al. . Features of microbiocenoses of various biotopes in women as a potential factor risk of miscarriage. Epidemiology and Vaccinal Prevention. 2021;20.

9. Grewal K, Lee YS, Smith A, Brosens JJ, Bourne T, Al-Memar M, et al. Chromosomally normal miscarriage is associated with vaginal dysbiosis and local inflammation. Bmc Medicine. 2022;20(1).

10. Gryaznova M, Kozarenko O, Smirnova Y, Burakova I, Syromyatnikov M, Maslov A, et al. Cervical and Vaginal Microbiomes in Early Miscarriages and Ongoing Pregnancy with and without Dydrogesterone Usage. International Journal of Molecular Sciences. 2023;24(18):13836.

11. Guang Y, Shen X, Tan Y, Tang SM, Chen J, Zhang LH, et al. Systematic analysis of microbiota in pregnant Chinese women and its association with miscarriage. Annals of Translational Medicine. 2022;10(20).

12. Han Y. Analysis of uterine microbiota in abortion and non-pregnant female based on high-throughput sequencing Journal of Shanghai Jiaotong University(Medical Science). 2019;12:165-9.

13. Jiao X, Zhang L, Du D, Wang L, Song Q, Liu S. Alteration of vaginal microbiota in patients with recurrent miscarriage. Journal of Obstetrics and Gynaecology. 2022;42(2):248-55.

14. Liu X, Cao Y, Xie X, Qin X, He X, Shi C, et al. Association between vaginal microbiota and risk of early pregnancy miscarriage. Comp Immunol Microbiol Infect Dis. 2021;77:101669.

15. Liu FT, Yang S, Yang Z, Zhou P, Peng T, Yin J, et al. An Altered Microbiota in the Lower and Upper Female Reproductive Tract of Women with Recurrent Spontaneous Abortion. Microbiol Spectr. 2022;10(3):e0046222.

16. Masucci L, D'Ippolito S, De Maio F, Quaranta G, Mazzarella R, Bianco DM, et al. Celiac Disease Predisposition and Genital Tract Microbiota in Women Affected by Recurrent Pregnancy Loss. Nutrients. 2023;15(1).

17. Moreno I VF, Martinez JF, Codoner FM, Ramon D. Impact of the Endometrial Microbiome on Uterine Receptivity and

Pregnancy Outcome. Reproductive Sciences. 2015;22:224A.

18. Moreno I, Codoñer FM, Vilella F, Valbuena D, Martinez-Blanch JF, Jimenez-Almazán J, et al. Evidence that the endometrial microbiota has an effect on implantation success or failure. Am J Obstet Gynecol. 2016;215(6):684-703.

19. Moreno I, Garcia-Grau I, Perez-Villaroya D, Gonzalez-Monfort M, Bahceci M, Barrionuevo MJ, et al. Endometrial microbiota composition is associated with reproductive outcome in infertile patients. Microbiome. 2022;10(1).

20. Mori R, Hayakawa T, Hirayama M, Ozawa F, Yoshihara H, Goto S, et al. Cervicovaginal microbiome in patients with recurrent pregnancy loss. Journal of Reproductive Immunology. 2023;157:103944.

21. Peuranpää P, Holster T, Saqib S, Kalliala I, Tiitinen A, Salonen A, et al. Female reproductive tract microbiota and recurrent pregnancy loss: a nested case-control study. Reprod Biomed Online. 2022;45(5):1021-31.

22. Seo SS, Arokiyaraj S, Kim MK, Oh HY, Kwon M, Kong JS, et al. High Prevalence of Leptotrichia amnionii, Atopobium vaginae, Sneathia sanguinegens, and Factor 1 Microbes and Association of Spontaneous Abortion among Korean Women. Biomed Res Int. 2017;2017:5435089.

23. Severgnini M, Morselli S, Camboni T, Ceccarani C, Laghi L, Zagonari S, et al. A Deep Look at the Vaginal Environment During Pregnancy and Puerperium. Front Cell Infect Microbiol. 2022;12:838405.

24. Shahid M, Quinlivan JA, Peek M, Castano-Rodriguez N, Mendz GL. Is there an association between the vaginal microbiome and first trimester miscarriage? A prospective observational study. Journal of Obstetrics and Gynaecology Research. 2022;48(1):119-28.

25. Shi Y, Yamada H, Sasagawa Y, Tanimura K, Deguchi M. Uterine endometrium microbiota and pregnancy outcome in women with recurrent pregnancy loss. J Reprod Immunol. 2022;152:103653.

26. Shu JJ, Lin SX, Wu Y, Zhu J, Gong D, Zou X, et al. A Potential Role for the Uterine Microbiome in Missed Abortions. Journal of Biological Regulators and Homeostatic Agents. 2022;36(4):1055-63.

27. Sun D, Zhao X, Pan Q, Li F, Gao B, Zhang A, et al. The association between vaginal microbiota disorders and early missed abortion: A prospective study. Acta Obstet Gynecol Scand. 2022;101(9):960-71.

28. Takimoto K, Yamada H, Shimada S, Fukushi Y, Wada S. Chronic Endometritis and Uterine Endometrium Microbiota in Recurrent Implantation Failure and Recurrent Pregnancy Loss. Biomedicines. 2023;11(9).

29. Vaughn SJ, Moreno I, Simon C, Lathi RB. Comparing the uterine microbiome in recurrent pregnancy loss to parous fertile controls. Fertility and Sterility. 2019;111(4):e17-e8.

30. Vomstein K, Reider S, Böttcher B, Watschinger C, Kyvelidou C, Tilg H, et al. Uterine microbiota plasticity during the menstrual cycle: Differences between healthy controls and patients with recurrent miscarriage or implantation failure. J Reprod Immunol. 2022;151:103634.

31. Wang L, Chen J, He L, Liu H, Liu Y, Luan Z, et al. Association between the vaginal and uterine microbiota and the risk of early embryonic arrest. Front Microbiol. 2023;14:1137869.

32. Xu LF, Huang LN, Lian CY, Xue HL, Lu YF, Chen XJ, et al. Vaginal Microbiota Diversity of Patients with Embryonic Miscarriage by Using 16S rDNA High-Throughput Sequencing. International Journal of Genomics. 2020;2020.

33. Zhang F, Zhang T, Ma Y, Huang Z, He Y, Pan H, et al. Alteration of vaginal microbiota in patients with unexplained recurrent miscarriage. Exp Ther Med. 2019;17(5):3307-16.

34. Zhao F, Chen Y, Gao J, Wu M, Li C, Wang Z, et al. Characterization of Vaginal Microbiota in Women With Recurrent Spontaneous Abortion That Can Be Modified by Drug Treatment. Front Cell Infect Microbiol. 2021;11:680643.
